# Health Coaching for Low Back Pain and Hip and Knee Osteoarthritis: A Systematic Review with Meta-Analysis

**DOI:** 10.1093/pm/pnac099

**Published:** 2022-07-01

**Authors:** Joanna Louise Prior, Giovana Vesentini, Jose Antonio Michell De Gregorio, Paulo H Ferreira, David J Hunter, Manuela L Ferreira

**Affiliations:** Faculty of Medicine and Health, Sydney Musculoskeletal Health, The Kolling Institute, School of Health Sciences, University of Sydney, Sydney, New South Wales, Australia; Department of Gynaecology and Obstetrics, Botucatu Medical School, UNESP—Univ Estadual Paulista, Botucatu, São Paulo, Brazil; Faculty of Medicine and Health, Sydney Musculoskeletal Health, The Kolling Institute, School of Health Sciences, University of Sydney, Sydney, New South Wales, Australia; The George Institute for Global Health, Sydney, Australia; Discipline of Physiotherapy, Faculty of Medicine and Health, University of Sydney, Sydney, New South Wales, Australia; Faculty of Medicine and Health, Sydney Musculoskeletal Health, The Kolling Institute, School of Health Sciences, University of Sydney, Sydney, New South Wales, Australia; Rheumatology Department, Royal North Shore Hospital, St Leonards, NSW 2065 Australia; Faculty of Medicine and Health, Sydney Musculoskeletal Health, The Kolling Institute, School of Health Sciences, University of Sydney, Sydney, New South Wales, Australia

**Keywords:** Low Back Pain, Osteoarthritis, Knee Pain, Hip Pain

## Abstract

**Background:**

Health coaching aims to empower people to reach their goals and is increasingly used in health care settings. Whether health coaching improves pain and disability for people with hip or knee osteoarthritis (OA) or low back pain (LBP) is unknown.

**Methods:**

Six databases were searched for randomized controlled trials assessing health coaching or motivational programs in adults with hip or knee OA or LBP, with each condition investigated independently. Meta-analyses were performed with random-effects models in the Cochrane Collaboration Review Manager 5.3 program.

**Results:**

Seventeen eligible studies were found. No studies analyzing hip OA alone were found. Pooled analyses found statistically significant decreases in mid-term pain (mean difference [MD]: –7.57; 95% confidence interval [CI]: –10.08 to –5.07; *P* < 0.001, *I*^2^ = 0%), short-term disability (standard mean difference [SMD]: –0.22; 95% CI: –0.41 to –0.03; *P* = 0.02, *z* = 2.32, *I*^2^ = 0%), and mid-term disability (SMD: –0.42; 95% CI: –0.75 to –0.09; *P* = 0.01, *z* = 2.49, *I*^2^ = 60%), favoring the intervention for chronic LBP. There were significant improvements in knee OA long-term functional disability (MD: –3.04; 95% CI: –5.70 to –0.38; *P* = 0.03; *z* = 2.24; *I*^2^ = 0%).

**Conclusion:**

Meta-analyses provide evidence that health coaching reduces both disability and pain in people with chronic LBP and reduces disability in people with knee OA, though the clinical significance is unknown. There is currently no evidence supporting or refuting the use of health coaching for hip OA.

## Introduction

The growth and aging of the world population has led to significant increases in the incidence and prevalence of lifestyle-related musculoskeletal disorders, such as low back pain (LBP) [1] and hip and knee osteoarthritis (OA) [2]. These conditions are the leading causes of adult musculoskeletal pain and economic burden worldwide [3]. The magnitude of this burden has been highlighted by the estimated global number of years lived with disability: 64.9 million associated with LBP, 8.3 million for knee OA, and 1.3 million for hip OA [4]. High prevalence has a direct impact on the financial burden of these conditions, with the annual Australian direct health care expenditure for LBP being AUD4.8 billion in 2013 [5]. Reported figures are likely to underestimate the true out-of-hospital expenditure because of the extensive use of over-the-counter pain relief medication and the use of private services, such as physiotherapy [6]. In comparison, the U.S. total direct and indirect costs attributed to LBP were estimated to be US$84.1 billion and US$624.8 billion [7]. Australian health care costs for OA in 2015 came to a total of AUD5.5 billion, with indirect costs estimated to be at least twice this value [8]. These costs are expected to continue to rise, and the burden on already overloaded health care systems is unsustainable. Finding ways to alleviate this pressure and reduce costs and dependency on health care systems while maintaining or improving health outcomes is therefore of paramount importance globally.

Lifestyle behaviors are primary contributors to chronic disease development and influence their progression and severity through noncompliance with recommended treatment [9]. Although they differ in anatomic position and function, LBP and knee and hip OA have similar guideline management recommendations that encompass nonpharmacological and pharmacological interventions, including education, advice to remain active, exercise, analgesics, and nonsteroidal anti-inflammatories [10–13]. Implementation of these guidelines in health care and patient adherence to evidence-based programs have been demonstrated to be suboptimal [14, 15].

In recent years, health coaching (HC) has been identified as a way of empowering individuals to self-manage their conditions. It draws on many behavior change theories, such as the “trans-theoretical model of change” and “motivational interviewing,” which aim to help patients remain healthy and prevent costly hospital admissions resulting from failing to follow recommended treatment plans [16]. A typical HC model involves identifying how an individual’s ideal health status differs from their current position and then exploring ways to close this gap [17]. With support, the individual works toward a more sustainable, health-promoting change in behavior [17]. The success of HC has been demonstrated in other chronic conditions, with increased patient adherence to physical exercise, weight loss programs, and recommended therapeutic regimes in hypertension and diabetes [18]. It has also been shown to help patients with cancer self-manage pain [19] and improve quality of life for patients with chronic obstructive pulmonary disease [20]. It is an area of increasing interest for LBP and hip and knee OA, with the hope that it can improve health outcomes and reduce costs, but to date, the effectiveness of HC for these conditions has been unclear.

The present review aimed to systematically appraise the literature investigating the effectiveness of HC to reduce pain and disability and inform on the recommended dosage for people with LBP and hip or knee OA.

## Methods

This systematic review was registered with PROSPERO, the International prospective register of systematic reviews (registration number CRD42018086072).

### Search Strategy

The search strategy was developed in collaboration with a research librarian and consisted of four separate components that used Medical Subject Headings (MeSH) and keywords (Supplementary Data Appendix 1). These were then combined with the HC component. The following databases were searched electronically up to November 24, 2021: Ovid MEDLINE^®^ >1946; Ovid EMBASE >1947; Cochrane Central Register of Controlled Trials; Ovid PsycINFO >1967; EBSCO CINAHL; and Web of Science. A hand search of the included studies’ reference lists and any relevant systematic reviews found was also conducted.

### Study Selection

Two independent reviewers (JLP and GV) screened all records by titles and then abstracts in accordance with the eligibility criteria. Full texts were obtained and screened, with any disagreement referred to a third independent reviewer (MLF) for resolution via a blinded vote count.

Randomized controlled trials published in peer-reviewed journals in any language were included if they contained at least one of the target populations, with participants 18 years of age or older and with symptoms of any duration. Diagnosis of knee or hip OA was in accordance with the American College of Rheumatology clinical criteria, radiographic diagnosis, or medical practitioner diagnosis. LBP studies were excluded if the target population included pregnancy-related LBP, serious spinal pathologies (e.g., fracture, malignancy, inflammatory disease), or LBP associated with neural tissue involvement (e.g., radiculopathy, spinal canal stenosis). To be included, studies needed to involve HC or motivational programs that aligned with HC models. Studies based on cognitive behavioral therapy were excluded because of the complex, structured nature of this psychological treatment that targets maladaptive thought patterns. Comparators could include usual care (e.g., general practitioner management or physiotherapy, including exercise, advice, manipulative therapy, or electrotherapy), no treatment, or any other conservative treatment. Although the registered protocol stated that only studies in English would be included, to minimize selection bias, the search was not limited by language, with Google Translate (Google LLC, Mountain View, CA, USA) used as required.

### Outcomes

Our primary outcomes were pain and disability. The secondary outcome measures of exercise compliance, physical activity, quality of life, sleep, weight loss, health care use, adverse events, and costs are listed in Supplementary Data Appendix 2.

### Data Extraction

Two reviewers (JLP and GV) independently extracted data from the selected full texts into a standardized data extraction spreadsheet. Disagreements were resolved by discussion between the two reviewers; the third reviewer was not required. Descriptive data pertaining to study setting, population (sample size, gender, age), and intervention characteristics (duration, number of sessions, content, mode of delivery, additional intervention components) were collected. For each study, relevant outcome measures were collected at each time point. Data were extracted as means with standard deviations (SDs). If only 95% confidence intervals (CIs) were available, SDs were calculated with the Cochrane Collaboration Review Manager (RevMan) 5.3 Software. Outcome data were extracted for the short-term (≤3 months), mid-term (>3 but <12 months), and long-term follow-ups (≥12 months).

### Methodological Quality Assessment

The methodological quality of included studies was assessed with the Physiotherapy Evidence Base Database (PEDro) scale [21]. This scale assesses the internal and external validity of studies and scores them via 11-point criteria. Studies were rated from 0 to 10, as the first criterion is excluded from the final score.

### Strength of the Body of Evidence

The strength of the body of evidence after meta-analysis was appraised with the GRADE approach (Grading of Recommendations Assessment, Development, and Evaluation) [22]. The quality of the evidence was rated down according to the following GRADE criteria: risk of bias (if 50% studies had low methodological quality that scored <7/10 on the PEDro scale); inconsistency (unexplained heterogeneity [*I*^2^ > 50%]); imprecision (small sample size [≤400 participants]); indirectness (participants, intervention, or outcomes differ from those of interest); and publication bias (industry-funded studies or all small studies included).

### Data Analysis

Studies were considered for meta-analysis if the comparisons were similar in terms of content, dosage, and follow-up time points. Meta-analyses were performed through the use of random-effects models in the Cochrane Collaboration Review Manager 5.3 program. Each condition was analyzed independently, with separate analyses performed for each outcome and time point where available.

Pain intensity measures were 0- to 100-mm visual analog scales or 0–10 numerical rating scales and were converted for the analysis to a common 0–100 scale. Results are presented as mean differences (MDs) with 95% confidence intervals (95% CIs). Different scales were used to measure disability (e.g., Roland-Morris Disability Questionnaire, Oswestry Disability Index), and data were pooled and presented as standardized mean differences (SMDs) with 95% CIs. Clinical significance was defined as an MD of at least 20 points on a 0- to 100-point scale [23]. An SMD of 0.2 was considered a small treatment effect, whereas 0.4 was considered a moderate and 0.8 a large treatment effect [24, 25]. Between-study statistical heterogeneity was assessed with the *I*^2^, with values <40% considered low heterogeneity, values of 40% to 75% moderate heterogeneity, and values >75% considerable heterogeneity [26].

## Results

### Study Selection

**Figure 1.**
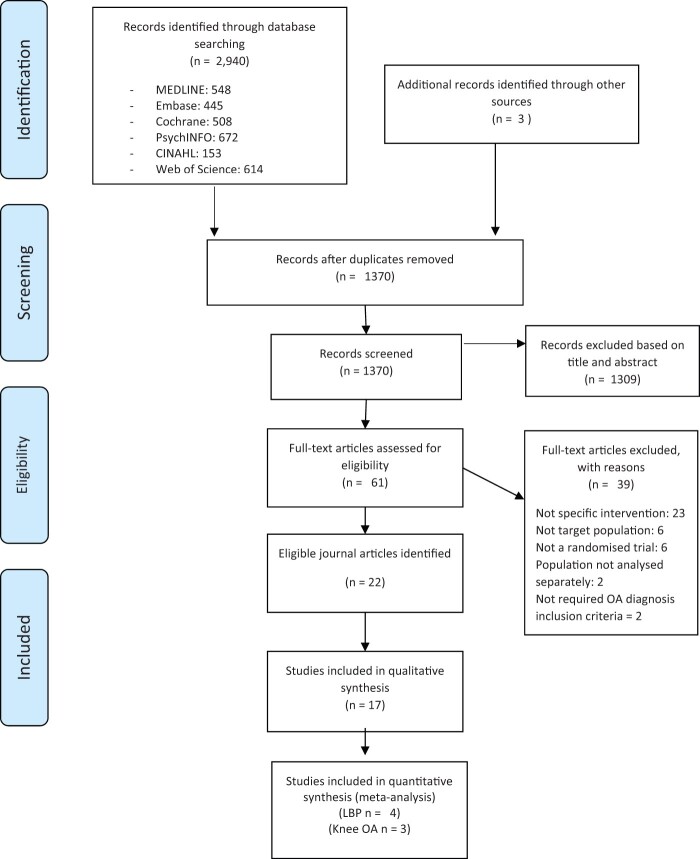
Consort PRISMA flow chart.

A total of 2,880 studies were identified. After removal of duplicates, 1,350 titles and 237 abstracts were screened by the two independent reviewers. A total of 61 studies were identified for full-text review; of those, 22 articles reporting on 17 randomized controlled trials (six studies on knee OA and 11 studies on LBP) were included in the review (Figure 1). Studies identified that required translation via Google Translate were subsequently excluded on the basis of the inclusion and exclusion criteria.

### Study Characteristics

Included studies were published between 1998 and 2020, with six studies conducted in Europe [27–32], six in Australia [33–38], three in North America [39–41], and two in Asia [42, 43]. One study used Zelen’s design, where participants were identified from private insurance company claims histories and randomly allocated to treatments before being invited into the study [30]. No studies were found in which hip OA was analyzed separately from other conditions. See Table 1.

**Table 1:** Study descriptions

Study	Inclusion Criteria	Sample Characteristics		Intervention Content	
		Control	Intervention	Control	Intervention
**Hip/knee**					
Bennell et al. 2017 [33] (Australia) Private clinics Intervention provider: nurses, occupational therapist, health psychologist	Age ≥50 years, average knee pain ≥4 on NRS, ACR clinical criteria for knee OA, classified as sedentary or insufficiently physically active according to the AAS.	n = 84 Age: 63.4 ± (7.8) Women: 58% Symptom duration: (%) >2 yrs 22 ± 26; 2–10 yrs 44 ± 52; >10 yrs 18 ± 21	n = 84 Age: 61.1 ± (6.9) Women: 68% Symptom duration: (%) >2 yrs 29 ± 35 2–10 yrs 39 ± 46 >10 yrs 16 ± 19	Five individual 30-minute physiotherapy sessions over 6 months. Physiotherapist provided verbal and written OA information and prescribed a progressive, individualized home exercise program. Patients were advised to complete the exercises 3×/week. They promoted increased general physical activity with a pedometer and optional self-monitoring/motivation.	**HealthChange methodology** Same physiotherapy intervention as control group; six telephone coaching sessions provided by a nurse over 6 months with up to six optional additional sessions.
Brosseau et al. 2012 Parts 1 [39] and Part 2 [45] (Canada) Community Intervention provider: unknown	Diagnosis mild–moderate unilateral/bilateral knee OA according to ACR clinical and radiographic/MRI criteria; pain for at least 3 months; expectation of medication change during study; able to walk a minimum of 20 minutes with pain (≤3/10 on VAS).	n = 79 (W) n = 74 (C) Age: 63.9 ± 10.3 (W); 62.3 ± 6.8 (C) Women: 69.9% (W); 63.5% (C) Symptom duration, years: 9.54 ± 8.1 (W); 10 ± 9.9 (C)	n = 69 Age: 63.9 ± 8.2 Women: 73.9% Symptom duration, years: 11.2 ± 9.7	**Walking (W):** 3×/week 65-minute supervised walking sessions for 12 months with a target intensity of 50–70% of individual’s predetermined maximum heart rate. Provision of a pedometer, a logbook, and an educational pamphlet. **Control (C):** Received an educational pamphlet, a pedometer, and a logbook.	The same intervention as the walking group. In addition, attended **PACEex behavioral program,** consisting of 20 educational sessions over 20 weeks, monthly face-to-face counseling for the first 6 months: support/encouragement to adhere to physical activity, barrier identification, self-management strategies to overcome barriers, goal setting. Followed by monthly telephone counseling for another 6 months for long-term goal planning and discussing barriers and facilitators to adherence to the walking program.
Gilbert et al. 2018 [40] (United States) Community Intervention provider: nurses, occupational therapists	Age ≥18 years, knee symptoms (pain, aching, or stiffness) on most days in prior month, able to ambulate at least 50 ft, radiographic knee OA defined by Kellgren–Lawrence ≥ Class 2.	n = 79 Age: 64.8 ± 12.4 Women: 62.03% Symptom duration, years: 12.2 ± 11.8	n = 76 Age: 61.41 ± 13.3 Women: 57.89% Symptom duration, years: 9.6 ± 8.3	One physician activity counseling session—involving determining whether patient self-reported 30 minutes moderate activity/day on most days and encouraged to work toward or maintain this level.	**Motivational interviewing** One physician activity counseling session as per control group. Initial face-to-face motivational interviewing session 45–60 minutes involving individual goal setting, tailored strategies to increase physical activity, identification of facilitators and barriers, progress monitoring. Subsequent sessions at 3, 6, and 12 months and 2 every 6 months in the second year for 10–15 minutes face-to-face or by phone.
Hinman et al. 2020 [35] (Australia) Community Intervention provider: Physiotherapists	Age ≥45 years, activity-related joint pain, ≤30 minutes morning stiffness, average knee pain ≥4/10, knee pain for at least 3 months.	n = 88 Age: 62.5 ± 8.1 Women: 63% Symptom duration: 9 ± 8 years	n = 87 Age: 62.4 ± 9.1 Women: 63% Symptom duration: 10 ± 9 years	One phone call for 25–40 minutes from a nurse, with follow-up calls if required. Provision of self-management information, verbal and written information, linkages to community resources, and/or referral to consumer and/or organizational websites.	**HealthChange methodology** The same program as the control. Additional survey for clinical history, physical limitations, and personal goals. Physiotherapist provides 5–10 phone calls over 6 months. Use a person-centered, collaborative approach to develop specific personalized self-management goals, a structured home strengthening exercise program, and a physical activity plan.
Li et al, 2020 [41] (Canada) Community Intervention providers: physiotherapists	Physician-confirmed knee OA, age ≥50 years, had pain/discomfort at knee lasting >28 consecutive/separate days, no prior history of inflammatory diseases or fibromyalgia; disease-modifying drugs; knee arthroplasty or awaiting total knee/hip surgery; acute knee injury in past 6 months	n = 25 Age: 64.8 ± 9 Women: 76% Symptom duration: NA	n = 26 Age: 65.0 ± 8 Women: 89% Symptom duration: NA	Received nothing for the first 14 weeks, then received the same program as the intervention group.	**Brief action planning and motivational interviewing:** Initial person session involving 20 minutes group education, 30 minutes of individual counseling, and education on the use of Fitbit Flex-2 wristband. Physiotherapist then provided biweekly phone counseling to modify physical activity goals for 8 weeks.
O’Brien et al. 2018 [37] (Australia) Community Intervention provider: university-qualified health coaches	Referred for outpatient orthopedic consultation, age ≥18 years, OA knee pain >3 months, overweight/obese (BMI ≥27 kg/m^2^ and <40 kg/m^2^), average knee pain ≥3/10 (NRS 0–10) in past week or moderate interference in activities of daily living, access to phone.	N = 60 Age: 60.2 ± 13.9 Women: 58% Symptom duration, days on waiting list: median (IQR): 390.0 (313.0–532.0)	N = 60 Age: 63.0 ± 11.1 Women: 66% Symptom duration, days on waiting list: median (IQR): 379.0 (279.0–507.0)	Telephone interview providing brief advice and education about the benefits of weight loss and physical activity. All remained on the waiting list to have an orthopedic consultation and could progress to surgery if recommended.	**Self Determination Theory** Same initial telephone interview as a control group. Additional referral to NSW Get healthy Information and coaching service for weight loss support, providing 10 telephone individually tailored coaching sessions over 6 months.
**LBP**					
Basler et al. 2007 [27] (Germany) Outpatient physiotherapy Intervention provider: physiotherapist	Minimum age 65 years, diagnosis of chronic LBP due to osteoporosis or degeneration with/out previous spine surgery and pain at the time of inclusion.	n = 84 Age: 70.6 ± 4.6 Women: 65.5% Symptom duration: NA	n = 86 Age: 70.1 ± 4.2 Women: 62.8% Symptom duration: NA	Ten individual physiotherapy sessions of 20 minutes over 5 weeks. Involved stretching and treatments tailored to improve trunk/lower limb length, strength, and endurance. Home program and written material to facilitate performance. Addition of 10 minutes of placebo ultrasound with an inactivated device.	**Trans-theoretical model of change** The same physiotherapy program as for the control. Addition of 10 minutes of counseling before each session by the treating physiotherapist.
Becker et al. 2008 [28] (Germany) Primary care Intervention provider: nurse	Nonspecific LBP on day of recruitment, older than 19 years.	n = 479 (GL) n = 410 (C) Age: 40.1 ± 13.3 (GL); 50.2 ± 14.3 (C) Women: 59.3% (GL); 52.9% (C) Symptom duration: NA	n = 489 Age: 47.4 ± 13.5 Women: 61% Symptom duration: NA	**GP Guideline Implementation (GL):** Participants received a multifaceted review by GP who had received training in the use of the LBP guideline of the DEGAM. **Control (C):** Participants reviewed by GPs who had received the LBP guideline through the post.	**Motivational interviewing** GPs received the same training as the GL group combined with training of the practice nurses in motivational interviewing. Nurses provided up to three counseling sessions of 10–15 minutes each and used specifically designed brochures and posters on motivation and behavior change according to the individual’s stage of change identified.
Friedrich et al. 1998 [29]/Friedrich et al. 2005 [46] (Austria) Hospital outpatients Intervention provider: physical therapists	Age 20–60 years with recurrent LBP with/out radiation to legs or LBP >4 months.	n = 49 Age: 44.9 ± 11 Women: 44.9% Symptom duration: NA	n = 44 Age: 43.3 ± 10.4 Women: 56.8% Symptom duration: NA	Ten sessions with a physical therapist lasting 25 minutes over 5 weeks. The exercise program consisted of individualized, submaximal exercises.	The same as for the control group. In addition, they received a 5-point motivational program: counseling involving education and enhancing patient’s internal locus of control, barrier identification, and problem solving; reinforcement techniques, consisting of positive therapist feedback and positive reinforcement; oral agreements and written treatment contracts; placement of agreement in prominent place at home; exercise diary.
Gardner et al. 2019 [34] (Australia) Primary care clinics and community settings Intervention provider: physiotherapist	Age 18–65 years, nonspecific LBP of minimum 3 months, pain ≥4/10, disability of ≥20 points on Quebec Back Pain Disability Scale	n = 37 Age: 45 ± 13.8 Women: 65.8% Symptom duration: 9.9 ± 8.7 years	n = 38 Age: 44 ± 12.5 Women: 48.6% Symptom duration: 10.3 ± 10.9 years	Three face-to-face sessions (at baseline 1 hour, 30 minutes, 2 and 4 months) with treating physiotherapist to discuss LBP history and give advice on a standardized exercise program. Sessions were designed for exercise review and participant retention.	**Patient-Led Goal Setting** Received a participant handbook containing neuroscience education, LBP information, self-management tips, goal setting information. Five face-to-face sessions with a physiotherapist: baseline 1 hour, four sessions (15–30 minutes) at 2-week intervals, then two follow-up sessions (30 minutes) 1 month apart. Final session was at 12 months from baseline to collect outcome measures. Sessions involved problem identification, prioritization, evidence -based strategies, patient-specific goals, and strategies toward goal achievement, barriers identified.
Hüppe et al. 2019 [30] Germany Private health insurer Intervention provider: unknown	Age ≥18 years, billed for at least two treatments for back pain and one/more cases of temporary work disability or two/more prescriptions of opioids or persistent mood disorder	n = 255 Age: 53.6 ± 8.7 Women: 40.4% Symptom duration: NA	n = 189 Age: 53.4 ± 8.1 Women: 31.7% Symptom duration: NA	Usual care, i.e., care according to the prescriptions of their health care providers (family doctors or medical specialists). Specific details were not available.	**HC** Initial physician review, tailored exercise program involving strength training, gymnastics, and relaxation exercises for 60 minutes/session up to 24 hours within 3–4 months. Personal HC via phone by external professional coach during treatment phase and 6 months aftercare. Up to 222 minutes over 16 sessions.
Iles et al. 2011 [36] (Australia) Physiotherapy outpatients Intervention provider: physiotherapist	Age 18–64 years with nonspecific LBP with onset within previous 8 weeks, low–moderate expectation of recovery.	n = 15 Age: 39.5 ± 12.7 Women: 33% Symptom duration: 25.1 days	n = 15 Age: 39.5 ± 11.7 Women: 47% Symptom duration: 25.5 days	Physiotherapy management at the discretion of the treating physiotherapist, including treatment type, frequency, and discharge, to ensure it reflected usual physiotherapy care (mean of 5.4 control sessions and 6.3 intervention sessions).	**Trans-theoretical model of change** Physiotherapy usual care as per control. In addition, 30-minute weekly HC via telephone for 4 weeks. Final session 3 weeks later. A total of five coaching calls were implemented, regardless of the continuation of physiotherapy.
Lonsdale et al. 2017 [31] (Ireland) Outpatient clinics Intervention provider: physiotherapists	Age 18–70 years, mechanical LBP with/out radiation to lower limb for >3 months or recurrent episodes in previous year.	n = 124 Age: 46.7 ± 13.5 Women: 52% Symptom duration: NA	n = 131 Age: 44.1 ± 13 Women: 56% Symptom duration: NA	Physiotherapy usual care. No limit on frequency or type of treatment given (mean of three sessions both groups).	**Self-determination theory model of behavior change** Same as for the physiotherapy usual care control except treatment was delivered by physiotherapists who had completed the CONNECT training.
Schaller et al. 2017 [32] Germany Inpatient rehabilitation center Intervention provider: sports scientist	Age 18–65 years, starting inpatient rehabilitation for LBP	n = 73 Age: 53.3 ± 6.3 Women: 37% Symptom duration: 82% >12 months	n = 71 Age: 50.4 ± 7.3 Women: 28% Symptom duration: 86% >12 months	Inpatient: two 30-minute lectures on health-enhancing physical activity Aftercare: able to download the lectures	**Rubicon’s model of Action Phases** Inpatient: ×2, 60-minute small group sessions focusing on motivation, perceived consequences of physical activity, self-efficacy beliefs, planning individual physical activity after rehabilitation, and barriers and solution strategies. Phone aftercare: ×2+ tailored phone calls reviewing current physical activity, barriers and facilitators, and future physical activity planning. Internet aftercare: 12-month access to specific information on LBP.
Thanawat and Nualnetr 2017 [42] (Thailand) Sub District Hospitals Intervention provider: unknown	Male/female, age 30–50 years, currently working on rice paddy field, at least two crops harvested annually for more than a year, nonspecific LBP on most days over at least 3 months, with/without having radiating pain in one/both legs, willing to participate and cooperate with the study procedures.	n = 64 Age: 44.7 ± 5.4 Women: 82.8% Symptom duration: NA	n = 62 Age: 45 ± 5.4 Women: 83.9% Symptom duration: NA	An 8-week intervention program including health education and exercises delivered every 2 weeks. A booklet and audio-visual materials were provided along with a home-based exercise program designed to strengthen the trunk. Monthly home visits to encourage home exercises and monitor symptoms for 6 months after the end of the 8-week program.	**Trans-theoretical model of change** The same 8-week intervention as the controls. However, strategies used to provide the intervention were different according to the identified stage of change for the individual.
Vong et al. 2011 [43] (Hong Kong) Outpatient physiotherapy Intervention provider: physiotherapist	Age 18–65 years with nonspecific LBP for at least 3 months	n = 38 Age: 45.1 ± 10.7 Women: 68.4% Symptom duration: 4.25 years	n = 38 Age: 44.6 ± 11.2 Women: 57.9% Symptom duration: 3.46 years	Ten physiotherapy sessions lasting 30 minutes over 8 weeks. Treatment involved 15 minutes of IFT and individually tailored back exercise program adopted from an exercise book. Exercises were prescribed for home, and participants were requested to exercise daily.	**Motivational interviewing** Same as the control. In addition, during the physiotherapy sessions, the intervention group received MET from the therapists, who integrated MI skills and psychological components designed to enhance motivation and make appropriate behavior changes.
Williams et al. 2018 [38] (Australia) Community Intervention provider: qualified health professional	Referred for outpatient orthopedic consultation, nonspecific LBP, average pain ≥3/10 in past week, moderate interference with ADL, age 18 years or over, overweight/obese (BMI ≥27 kg/m^2^), phone access.	n = 80 Age: 57.4 ± 13.6 Women: 66% Symptom duration: 18.5 ± 15.7 years	n = 79 Age: 56.0 ± 13.3 Women: 60.8% Symptom duration: 13.0 ± 11.9 years	Continued the usual care pathway (i.e., remained on the waiting list to have an orthopedic specialist consultation and could progress to consultation if scheduled) and took part in data collection during the study period. No other active intervention.	**Self-Determination Theory** Initial face-to-face consult with physiotherapist (up to 1 hour)—involved clinical assessment, back pain education, and advice and behavior change techniques. Referral to NSW Get Healthy telephone coaching, providing individually tailored sessions over 6 months using information on national healthy eating and physical activity guidelines. Used intention formation, setting graded tasks and specific behavior goals, barrier identification, and self-monitoring of behavior and outcomes.

AAS = Active Australia Survey; ACR = American College of Rheumatology; ADL = activities of daily living; BMI = body mass index; CONNECT = Communication Style and Exercise Compliance in Physiotherapy; DEGAM = German College of General Practitioners and Family Physicians; GP = general practitioner; IFT = Interferential therapy; IQR = interquartile range; MET = Motivational enhancement therapy; MI = Motivational interviewing; MRI = magnetic resonance imaging; NA = not applicable; NRS = numeric rating scale; VAS = visual analog scale.

### Intervention

#### Knee OA

HC dosage varied considerably, ranging from six sessions over 6 months [33] to nine sessions over 2 years [40], and additional intervention components differed extensively among studies. One study compared the addition of six HC telephone sessions and five additional physiotherapy sessions vs five physiotherapy sessions alone [33]. The second study compared an intensive supervised walking program (65 minutes, three times a week for 1 year) vs the same program with an additional 20 education sessions, six group counseling sessions, and 12 monthly phone counseling sessions, vs the provision of an information pamphlet alone [39]. Two studies compared one session of counseling vs 8–10 telephone HC sessions over 6 to 24 months [37, 40]. One study compared the use of a Fitbit (Fitbit, San Francisco, CA, USA) device vs four individual HC calls over an 8-week time frame vs no treatment [41]. The final study compared one or more sessions of an existing nurse-led phone education program vs 5–10 HC calls over 6 months [35] (Table 1).

#### Low Back Pain

HC provided in the LBP studies was generally of shorter duration but greater intensity than that provided in knee OA studies. HC dosage varied from one to three sessions [28, 31] to as many as 10 sessions over a 5- to 8-week period [27, 29, 43]. Two studies provided HC for 6 months, ranging from 10 to 16 sessions [30, 38]. Five studies incorporated HC into the usual physiotherapy sessions [27, 29, 31, 42, 43]. Four studies conducted HC remotely via telephone [30, 32, 36, 38]. One of these conducted an initial face-to-face physiotherapy session to provide back-specific education, advice, and behavioral change techniques, with all other contacts occurring via telephone [38]. Another provided inpatients with two small-group sessions followed by two or more HC calls once they were outpatients [32]. In most studies, physiotherapists provided the HC (Table 1).

### Underpinning Theories

A range of underpinning theories were used, with motivational interviewing [40, 41, 43, 44], the trans-theoretical model [27, 36, 42], and self-determination theory [31, 37, 38] used most frequently (Table 1). Three studies failed to report the underpinning theory [29, 30, 39], but the content description aligned with key components of HC for behavioral change.

### Methodological Quality of the Studies

The methodological quality of the included studies is shown in Table 2. All studies used random allocation of participants, with concealed allocation in 11 studies [27, 28, 30, 32, 33, 35–38, 40, 41]. Baseline characteristics were similar in all studies, except one that used Zelen’s design in which participants were randomized before consent, but analyses were adjusted for baseline differences [30]. PEDro scores ranged from 5/10 to 8/10, with an average PEDro score of 6.71 (SD: 1.05). The overall quality of the studies was considered to be moderate.

**Table 2.** Quality of the studies on the PEDro scale

STUDY	1. Eligibility criteria specified	2. Subjects were randomly allocated to groups	3. Allocation was concealed	4. Groups were similar at baseline	5. Blinding of subjects	6. Blinding of therapists	7. Blinding of assessors	8. Measures of key outcome were obtained from > 85% subjects	9. Data analysed by “intention to treat”	10. Between-group statistical comparisons reported	11. Point measures and measures of variability	TOTAL (/10)
**KNEE**												
Bennell et al. 2017 [33]	✓	✓	✓	✓	X	X	✓	✓	✓	✓	✓	8
Brosseau et al. Part 1 2012 [39]	X	✓	X	✓	X	X	✓	X	✓	✓	✓	6
Gilbert et al. 2018 [40]	✓	✓	✓	✓	X	X	✓	✓	X	✓	✓	7
Hinman et al. 2020 [35]	✓	✓	✓	✓	X	X	✓	✓	✓	✓	✓	8
Li et al. 2020 [41]	✓	✓	✓	✓	X	X	✓	✓	✓	✓	✓	8
O’Brien et al. 2018 [37]	✓	✓	✓	✓	X	X	✓	X	✓	✓	✓	7
**LBP**												
Basler et al. 2007 [27]	✓	✓	✓	✓	X	X	✓	✓	✓	✓	✓	8
Becker et al. 2008 [28]	✓	✓	✓	✓	X	X	X	✓	X	✓	✓	6
Friedrich et al. 1998 [29]	✓	✓	X	X	X	X	X	✓	✓	✓	✓	5
Gardner et al. 2019 [34]	✓	✓	X	✓	X	X	✓	✓	✓	✓	✓	7
Hüppe et al. 2019 [30]	✓	✓	✓	✓	X	X	X	X	✓	✓	✓	6
Iles et al. 2011 [36]	✓	✓	✓	✓	X	X	X	✓	✓	✓	✓	7
Lonsdale et al. 2017 [31]	✓	✓	X	✓	X	X	✓	X	✓	✓	✓	6
Schaller et al. 2017 [32]	X	✓	✓	X	X	X	X	X	✓	✓	✓	5
Thanawat and Nualnetr 2017 [42]	✓	✓	X	✓	X	X	X	✓	✓	✓	✓	6
Vong et al. 2011 [43]	✓	✓	X	✓	X	X	✓	X	✓	✓	✓	6
Williams et al. 2018 [38]	✓	✓	✓	✓	X	X	✓	✓	✓	✓	✓	8

### Intervention Effectiveness

The results of individual studies are listed in Supplementary Data Appendix 2. Meta-analyses were considered possible for knee OA pain and disability at mid- and long-term follow-up (see Figures 2, 3, 6, and 7) and chronic LBP pain and disability at short- and mid-term follow-up (see Figures 4, 5, 8, and 9).

**Figure 2.**
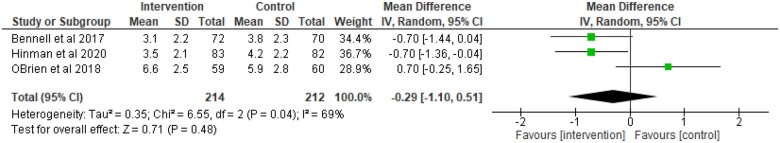
Pooled effect of intervention compared with control for mid-term pain levels for knee OA on numeric rating scale 0–100.

**Figure 3.**
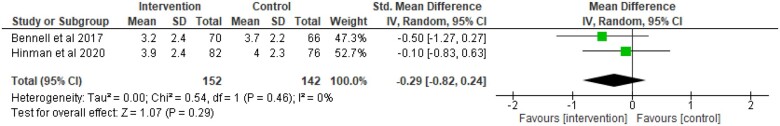
Pooled effect of intervention compared with control for long-term pain levels for knee OA on numeric rating scale 0–100.

**Figure 4.**
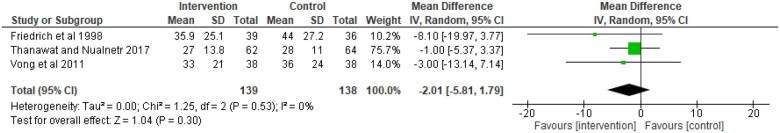
Pooled effect of intervention compared with control for short-term pain levels for chronic LBP on numeric rating scale 0–100.

### Strength of the Body of Evidence

The studies included in the meta-analyses were rated according to the GRADE criteria [22] in Table 3. The quality of evidence for LBP was considered very low for short-term pain and short-term to mid-term pain and disability and low for short-term disability. The evidence was marked down for risk of bias (50% studies of low methodological quality with individual PEDRo scores <7/10); imprecision (pooled analyses with combined sample sizes of ≤400); and suspected publication bias, as evidenced by the small available study sample sizes and absence of published study protocols or clinical trial registrations. The quality of the evidence was rated low for disability at short-term follow-up, as described above, but was not marked down for imprecision.

**Table 3.** Summary of the scoring using the GRADE system

Assessment of Overall Study Quality							Summary of Findings			
							Number of Participants		Effects (95% CI)	Certainty of Evidence
Studies and Time Point	Design	Risk of Bias	Inconsistency	Imprecision	Indirectness	Publication Bias	Control	Intervention		
**Pain**										
Chronic LBP short term 3 studies [29, 42, 43]	RCT	All studies <7/10 PEDro score Serious limitation	*I* ^2^ = 0% No serious limitation	Sample size ≤400 Serious limitation	Not detectable	Suspected	138	138	MD: –1.58 (–5.38 to 2.22) *I* ^2^ = 0% *P* = 0.52	Very low ⊕ΟΟΟ
Chronic LBP mid term 2 studies [29, 42]	RCT	All studies <7/10 PEDro score Serious limitation	*I* ^2^ = 0% No serious limitation	Sample size ≤400 Serious limitation	Not detectable	Suspected	103	103	MD: –7.57 (–10.11 to –5.03) *I* ^2^ = 0% *P* = 0.93	Very low ⊕ΟΟΟ
Knee OA mid term 3 studies [33, 35, 37]	RCT	All studies ≥7/10 No serious limitation	*I* ^2^ = 0% No serious limitation	Sample size ≤400 Serious limitation	Not detectable	Suspected	204	192	MD: –0.54 (–0.97 to –0.11) *I* ^2^ = 0% *P* = 0.01	Low ⊕⊕ΟΟ
Knee OA long term 2 studies [33, 35]	RCT	All studies ≥7/10 No serious limitation	*I* ^2^ = 0% No serious limitation	Sample size ≤400 Serious limitation	Not detectable	Suspected	142	152	MD: –0.29 (–0.82 to 0.24) *I* ^2^ = 0% *P* = 0.29	Low ⊕⊕ΟΟ
**Disability**										
Chronic LBP short term 4 studies [27, 29, 42, 43]	RCT	Most studies <7/10 PEDro score Serious limitation	*I* ^2^ = 46% No serious limitation	Sample size >400 No serious limitation	Not detectable	Suspected	224	222	SMD: 0.01 (–0.25 to 0.27) *I* ^2^ = 45% *P* = 0.14	Low ⊕⊕ΟΟ
Chronic LBP mid term 3 studies [27, 29, 42]	RCT	All studies <7/10 PEDro score Serious limitation	*I* ^2^ = 59% Serious limitation	Sample size ≤400 Serious limitation	Not detectable	Suspected	187	189	SMD: –0.05 (–0.70 to 0.59) *I* ^2^ = 89% *P* = 0.0001	Very low ⊕ΟΟΟ
Knee OA mid-term 3 studies [33, 35, 37]	RCT	All studies ≥7/10 No serious limitation	*I* ^2^ = 58% Serious limitation	Sample size ≤400 Serious limitation	Not detectable	Suspected	204	192	MD: –1.79 (–5.63 to 2.04) *I* ^2^ = 58% *P* = 0.36	Very low ⊕ΟΟΟ
Knee OA long term 2 studies [33, 35]	RCT	All studies ≥7/10 No serious limitation	*I* ^2^ = 0% No serious limitation	Sample size ≤400 Serious limitation	Not detectable	Suspected	142	152	MD: –3.04 (–5.70 to –0.38) *I* ^2^ = 0% *P* = 0.03	Low ⊕⊕ΟΟ

The quality of evidence for knee OA was considered low for pooled analyses on pain and long-term disability and very low for the pooled analysis of mid-term disability. The evidence was marked down because of imprecision (meta-analyses contained combined sample sizes ≤400), and publication bias was not detected. The pooled analysis on disability at the mid-term follow-up was also marked down because of inconsistency (unexplained heterogeneity [*I*^2^ > 50%]).

### Pain

#### Knee OA

All studies (n = 891) assessed pain, with a meta-analysis performed on three studies (n = 463) at mid- and long-term follow-up. These studies compared 5–10 sessions of HC over 6 months to existing physiotherapy or advice, education, and physical activity guidance [33, 35, 37]. The results show that the addition of HC provided no significant improvement in pain at mid-term follow-up (Figure 2; MD: –0.29; 95% CI: –1.10 to 0.51; *P* = 0.48; *z* = 0.71; *I*^2^ = 69%) or at long-term follow-up (Figure 3; MD: –0.29; 95% CI: –0.82 to 0.24; *P* = 0.29; *z* = 1.07; *I*^2^ = 0%).

A short-term, statistically significant difference favoring HC was found in one study providing baseline physician recommendation and nine HC calls over 2 years (MD: –0.97; 95% CI: –1.77 to –0.17; *P* = 0.02) vs physician recommendation alone [40]. The other studies found no difference between groups at any time point [41, 45]. Studies were excluded from the meta-analysis because of incomparable intervention dosages [40, 41] or incomparable additional therapies provided [39].

#### Low Back Pain

**Figure 5.**
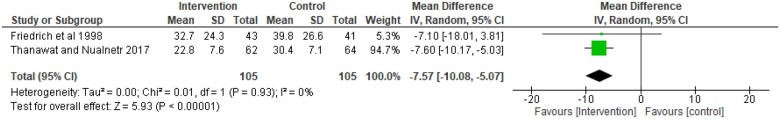
Pooled effect of the intervention compared with control for mid-term pain levels for chronic LBP on numeric rating scale 0–100.

Eight studies (n = 2,606) assessed pain [28–32, 38, 42, 43]. Short-term outcomes of three studies (n = 295) that compared physiotherapy vs physiotherapy plus HC / motivational strategies for chronic LBP were pooled [29, 42, 43]. The results show that HC added to usual physiotherapy care provided no further benefit compared with physiotherapy alone for pain reduction in the short term (Figure 4; MD: –2.01; 95% CI: –5.81 to 1.79; *P* = 0.30, *z* = 1.04; *I*^2^ = 0%). Pooled analysis of mid-term follow-up (two studies; n = 219), however, found the addition of HC to usual physiotherapy care resulted in a small but statistically significant effect on pain reduction compared with usual physiotherapy alone (Figure 5; MD: –7.57; 95% CI: –10.08 to –5.07; *P* = <0.001, *z* = 5.93, *I*^2^ = 0%). Three studies provided longer follow-up data (12 months [34], 24 months [30], and 5 years [46]), but no meta-analysis could be performed because of incompatible outcome measures (pain intensity vs number of days in pain) and different time points. One that compared HC and physiotherapy vs physiotherapy alone [29] found continued significant improvements in pain at 12 months compared with the control (MD: –15.50; 95% CI: –27.82 to –3.18; *P* = 0.02). Data collected at 5-year follow-up showed that the intervention group continued to have significantly less pain than at baseline, whereas the control group did not [46]. Reasons studies were excluded from the meta-analysis included having a shorter intervention length [27, 31], including acute LBP [36], or using interventions that were significantly different [30, 38].

### Disability

#### Knee OA

**Figure 6.**
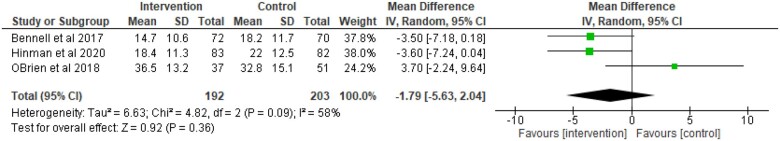
Pooled effect of the intervention compared with control for mid-term functional disability for knee OA.

**Figure 7.**
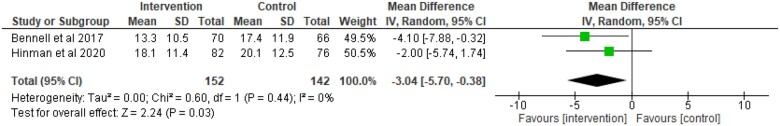
Pooled effect of the intervention compared with control for long-term functional disability for knee OA.

Five of the studies (n = 840) reported functional disability according to the Western Ontario and McMaster Universities Osteoarthritis Index function subscale [33, 35, 37, 40, 45]. The mid-term outcomes of three studies [33, 35, 37] (n = 463) and long-term outcomes of two studies [33, 35] (n = 343) were pooled. The results show that HC provided no significant improvement in disability at the mid-term follow-up (Figure 6; MD: –1.79; 95% CI: –5.63 to 2.04; *P* = 0.36, *z* = 0.92; *I*^2^ = 58%), but long-term follow-up showed a significant improvement in disability favoring HC (Figure 7; MD: –3.04; 95% CI: –5.70 to –0.38; *P* = 0.03, *z* = 2.24; *I*^2^ = 0%). In the studies not pooled, one compared eight motivational interviewing sessions vs one physician counseling session to encourage walking 30 minutes most days and found that the intervention group significantly improved in functional ability at 12 months compared with the control (MD: –3.19; 95% CI: –6.18 to –0.20; *P* = 0.04) [40]. The other study found no difference in disability between groups at any time point [45].

#### Low Back Pain

**Figure 8.**
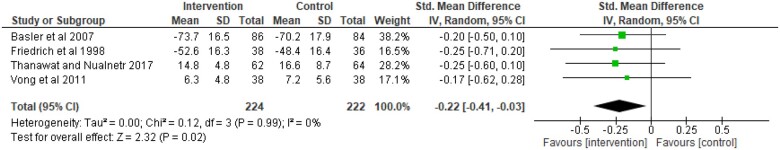
Pooled effect of the intervention compared with control for short-term disability for chronic LBP.

**Figure 9.**
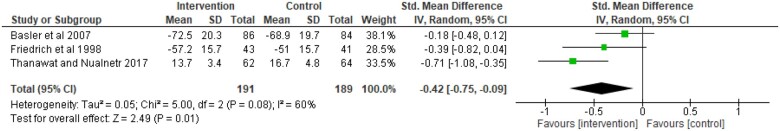
Pooled effect of the intervention compared with control for mid-term disability for chronic LBP.

All but one [32] of the LBP studies reported on disability (n = 2,806). Short-term results of four studies (n = 446) [27, 29, 42, 43] and mid-term results of three studies [27, 29, 42] (n = 376) were pooled in meta-analyses. The pooled results showed a small but significant improvement favoring HC in addition to usual physiotherapy care vs physiotherapy alone at short-term follow-up (Figure 8; SMD: –0.22; 95% CI: –0.41 to –0.03; *P* = 0.02, *z* = 2.32, *I*^2^ = 0%) and mid-term follow-up (Figure 9; SMD: –0.42; 95% CI: –0.75 to –0.09; *P* = 0.01, *z* = 2.9, *I*^2^ = 59%).

In the studies excluded from the meta-analysis, three studies found no difference in disability at short- or mid-term follow-up [31, 36, 38]. One found a significant improvement at 6 months in the group who received motivational interviewing and saw a guideline-trained general practitioner compared with the control group (MD: 3.650; 95% CI: 0.320 to 6.979; *P* = 0.032; scale 0–100) [28]. One study found a small but significant improvement in disability favoring HC at 24 months (MD: −0.24; 95% CI: −0.43 to −0.05; *P* = 0.025; scale 0–6) after a tailored exercise program with 16 telephone HC sessions over 6 months vs usual care [30]. The final study provided patient-led goal setting vs a standard exercise program and found significant improvements at each time point in favor of the intervention (2 months MD: 11.1; 95% CI: 3.8 to 18.3; *P* < 0.05; 4 months MD: 12.9; 95% CI: 5.6 to 20.1; *P* < 0.05; 12 months MD: 11.6; 95% CI: 3.6 to 19.5; *P* < 0.05; scale 20–100) [34].

### Secondary Outcomes

No significant differences were found in any other secondary outcome that could be pooled in a meta-analysis at any time point for either knee OA or LBP. Only one study reported a significant improvement in quality of life for LBP at 12 months [28], and one study reported a significant increase in the number of days on which participants exercised [30] favoring the intervention.

Only two studies reported the cost and savings for the intervention [35, 47]. One knee OA study reported that the direct cost of the intervention in addition to the existing nurse-led support was AUD514, with no evidence of savings in other health service resources [35]. One LBP study provided an economic evaluation of the intervention [47] and reported a mean intervention cost of AUD708 per person. Although that study found no difference between the intervention and control groups for any clinical outcomes (pain, disability, weight, or body mass index), the study found significantly lower costs in terms of health care services used for LBP (AUD292; 95% CI: −872 to −33), medication costs (AUD30; 95% CI: −65 to −4), and work absenteeism (AUD1,000; 95% CI: −3,573 to −210) favoring the intervention group [50]. The incremental cost-effectiveness ratios for quality-adjusted life years indicated that one quality-adjusted life year gained was associated with a societal cost saving of $31,087, demonstrating that the intervention was on average less costly and more effective than usual care [47]. Society’s willingness to pay per unit of effect gained was not determined [47].

## Discussion

The aim of this systematic review was to investigate the effect of HC on pain and disability for knee OA, hip OA, and LBP. A comprehensive search strategy across multiple databases identified 17 original studies. No study was found that included participants with hip OA that performed subgroup analysis of the individual musculoskeletal condition. Consequently, there is currently no evidence for the effect of HC specifically for the population with hip OA.

### Clinical Implications

The pooled results suggest that HC could have favorable outcomes for both the chronic LBP and knee OA populations at different time points, though the level of evidence remains low to very low. As the CIs crossed the pre-determined threshold for clinical significance for both disability and pain (i.e., an MD of 20 points on a 0- to 100-point scale [23] and an SMD of 0.2 [25]), we conclude that it is uncertain whether HC provides small but clinically important benefits for disability or pain (see Table 4 for assistance in the interpretation of effect sizes expressed as an SMD). Moreover, we lack information on the long-term effect and whether this strategy works better alone or in combination with other active management strategies.

**Table 4.** Interpretation of LBP disability effect size (SMD) expressed in original outcome measure scale

Outcome Measure	Scale	Short-Term SMD	Short-Term SMD Expressed on Original Scale	Mid-Term SMD	Mid-Term SMD Expressed on Original Scale
Hanover Functional Disability Questionnaire	0–100 0 = minimal function 100 = optimal function	–0.22	–3.785 points	–0.42	–8.4 points
Low Back Outcome Scale Questionnaire	0–78 0 = a great deal of disability 78 = no disability	–0.22	–3.597 points	–0.42	–6.59 points
Oswestry Disability Index Scale	0–100 0 = no disability 100 = severely disabled	–0.22	–1.53 points	–0.42	–1.752 points
Roland-Morris Disability Questionnaire	0–24 0 = no disability 24 = maximal disability	–0.22	1.15 points	–0.42	NA

NA = not applicable.

Only two studies reported on health service costs after the provision of HC alone for LBP [47] and knee OA patients on a specialist waiting list [37]. No cost savings were found for the population with knee OA [37]. Conversely, significant savings were found for the population with LBP [47], which might be more meaningful and crucial to the health care system for this population and requires further investigation.

Six LBP studies [27, 29, 31, 36, 42, 43] and one on knee OA [33] reported small between-group differences favoring HC in addition to physiotherapy vs physiotherapy alone, suggesting that adding HC to active interventions might not result in substantial additional improvements. Interestingly, two studies compared HC vs minimal care in patients with LBP [38] and knee OA [37] with high body mass index on an orthopedic waiting list. The studies found no difference between groups in any outcome at any time point except for the mental health component of quality of life at 6-month follow-up in participants with knee OA. The authors of these studies acknowledged the use of “non–disease-specific healthy lifestyle intervention,” though training for LBP and knee OA was given to the health coaches providing the intervention [37, 38]. They further identified that to adequately support and manage pain and facilitate lifestyle changes in the populations with chronic LBP and knee OA, a more intensive, disease-specific telephone intervention might be required [37, 38].

It is important to acknowledge that HC was usually found to be delivered simultaneously with physiotherapy management for LBP [27, 29, 31, 34, 36, 42, 43]. Providing HC when adherence levels tend to drop after completion of physiotherapy could prove more effective by providing greater accountability and support for self-management. A pilot study reviewed the use of HC for consistent pain after completion of hospital-based physiotherapy to reduce care-seeking, pain, and disability [48]. It found that HC was well accepted by patients and might help to reduce care-seeking in this population, but the outcome data could not be analyzed because the study was underpowered for such evaluation. Studies on knee OA delivered HC with a diverse range of simultaneous management strategies, including physiotherapy, nurse-led support, supervised walking programs, and physical activity tracking devices. Despite the range of additional management strategies, the present review has shown that the addition of HC could also be of benefit to this population.

Optimal HC dosage continues to require further clarification and standardization. Previous systematic reviews for telephone-delivered physical activity and dietary behavior change found that 12 or more phone calls over a 6- to 12-month period have been shown to improve behavior [18, 49]. Studies in the present review reporting significant improvements in pain favoring HC varied significantly in their frequency and duration for both LBP and knee OA. The programs for LBP, though intense, might have been too short, and some of the knee OA programs might have lacked effective intensity. Consequently, no recommendations on optimal frequency and duration for either population can be made. The pooled knee OA studies, however, provided 5–10 HC sessions over a 6-month period, which could help inform future knee OA HC studies and clinical programs.

All the knee OA studies used telephone-based HC, with only four LBP studies favoring this delivery mode [30, 32, 36, 38], despite telephone-based interventions demonstrating the potential to provide greater patient access and overcome barriers to accessing care, including time and travel requirements for face-to-face appointments [50]. Their value has been further highlighted during the global coronavirus disease 2019 (COVID-19) pandemic. A previous report suggested that telephone counseling hampered the ability to establish patient rapport [33], yet provision of face-to-face consultations via online methods might alleviate this issue. A proof-of-concept study offered fortnightly motivational interviewing via videoconference to participants with knee OA to improve physical activity [51]. That study (n = 61) found significant improvements in the intervention group in moderate to vigorous physical activity, daily steps, Knee Injury and Osteoarthritis Outcome Score activity of daily living subscale score, and quality of life in the short term. Despite this apparent favorable finding, long-term effects of delivering this type of intervention via videoconferencing remain unknown.

### Comparison with Previous Literature

To the present authors’ knowledge, there have been three previous systematic reviews in this area. The first was limited to motivational interviewing and included general musculoskeletal conditions (including fibromyalgia, osteoporosis, and LBP) [52]. The authors of that review identified the need for further well-designed randomized controlled trials to measure effectiveness. The second reviewed HC for LBP and concluded that there was a small and inconsistent body of literature supporting HC for this population [53]. The third reviewed the effect of HC delivered by physical therapists to patients with a range of disorders (LBP, diabetes, coronary artery disease, Parkinson’s disease, rheumatoid arthritis, and stroke) and found that HC had positive effects on physical activity in 6 of the 11 included studies [54]. Because of the heterogeneity of the studies, however, no meta-analysis could be performed. The secondary outcomes in this present review suggest that the addition of HC to usual physiotherapy care might result in improved exercise compliance in the population with LBP in the short [31, 43] and middle term [29] and in the population with knee OA in the short term [45] (Results in online appendix). These results align with a previous systematic review that showed improved compliance with therapeutic regimes in other non-musculoskeletal conditions [18]. Our systematic review identified seven additional LBP studies [29–32, 34, 38, 42] and a total of 13 studies not previously included in reviews [29–35, 37–42]. All studies in our review were of moderate to high methodological quality, and meta-analyses have provided more precise pooled estimates of treatment effect.

### Strengths and Limitations

Strengths of the present review include the extensive search strategy across multiple databases, followed by hand searches of included studies and relevant systematic reviews, with thorough screening and data extraction by independent reviewers who followed a pre-registered protocol. Alerts from each database were set to ensure the literature search was valid up to the date of acceptance for publication. We were also able to identify a larger number of studies involving comparable populations that enabled the pooling of data.

The limitations of this review should also be acknowledged. First, despite the extensive search involving multiple databases, it is possible that some studies were missed. The incompatible outcome measures used in the included studies (e.g., number of days in pain vs pain levels, differences in dosage, and additional interventions) prevented the remaining studies from being included in the meta-analysis. In addition, although the minimum number of studies with which to perform a meta-analysis was met [55], the studies included in the meta-analyses contained small participant numbers, meaning that they were potentially underpowered, with the possibility of producing a false negative result and therefore a Type II error [56].

Finally, there is no standardized content for HC interventions within the health care setting, and the studies in this review used numerous underpinning theories. Consequently, the content of the interventions differed. This limitation, however, is not dissimilar to interventions included in other systematic reviews, such as exercise for LBP [57] and lower limb OA [58]. Exercise programs in these reviews varied significantly and included individual and group programs, specific muscle strengthening, global strengthening, graded activity programs, and the addition of advice or behavioral techniques [57, 58]. It is likely that the differing intervention strategies in the studies included in the present review reflect differences that occur in real practice. There was also heterogeneity in the content of the control or comparator program in each study. Differences in the “usual physiotherapy care” given, whether this involved active or passive strategies or advice, also reflect the differences that occur in managing these conditions in real practice.

As previously found, few studies adequately described the training of the health professionals delivering the intervention, preventing replication [52, 53]. Assessments of treatment fidelity were reported in only three studies [27, 31, 33]. Holden et al. [53] observed that the efficacy of treatment was difficult to assess because of the lack of accurate treatment fidelity assessment, which continues to be valid in the present review. Future randomized controlled trials need to adequately report intervention content, training of professionals delivering the intervention, and treatment fidelity measures.

The authors of the present review acknowledge that the use of Google Translate for studies not published in English was likely open to misinterpretation and errors in translation that might have resulted in some studies being incorrectly excluded from this review.

### Conclusion

There is evidence that HC could provide pain relief in the middle term and improvements in disability in the short and middle term for patients with chronic LBP. Although the body of literature for knee OA remains small, there is low-level evidence that HC could provide improvements in disability in the long term. Studies evaluating the effects of HC for hip OA are notably lacking. To date, evidence appears unclear as to whether improvements from adding HC to management programs is clinically important.

## Authors’ Contributions

JLP: protocol design, database search, identification of eligible studies, data extraction, assessments of quality and analysis of results, extracting relevant data and conducting quality assessments, synthesizing results, and authoring of manuscript original draft. GV: duplicated eligibility screening, data extraction, and quality assessments. JAM: synthesis and interpretation of results. PHF: interpretation of results. MLF: review inception, protocol development, and interpretation of results. All authors reviewed prior drafts of the systematic review, discussed and commented on the results, and contributed to the manuscript.

## Supplementary Material

pnac099_Supplementary_Data

## Notes

Funding source: This review was funded by Medibank Better Health Foundation.

Study registration: PROSPERO, registration number CRD42018086072.
