# Supplementary material for: Health Coaching for Low Back Pain and Hip and Knee Osteoarthritis: A Systematic Review with Meta-Analysis
Source: Pain Med. 2022 Jul 1;24(1):32–51. doi: 10.1093/pm/pnac099 (PMC9825146; doi:10.1093/pm/pnac099)

**APPENDIX 1: Full search strategy**

Database: Ovid MEDLINE(R) <1946 to November Week 4 2017>

Search Strategy:

--------------------------------------------------------------------------------

1 health behavior/ or patient compliance/ (102310)

2 ((health* or wellness or lifestyle or life style) adj3 (behavio?r or coach*)).mp. (59635)

3 directive counseling/ or motivational interviewing/ (3528)

4 (motivation* adj3 (counsel* or interview*)).mp. (3571)

5 (behavio?r adj3 change*).mp. (26389)

6 Motivation/ (62626)

7 ((self or intrinsic* or internal*) adj3 motivat*).mp. (3864)

8 coach* psycholog*.mp. (7)

9 ((trans-theoretic* or thranstheoretic*) and model*).mp. (61)

10 Self Care/ (32089)

11 (self manag* or selfmanag*).mp. [mp=title, abstract, original title, name of substance word, subject heading word, keyword heading word, protocol supplementary concept word, rare disease supplementary concept word, unique identifier, synonyms] (13042)

12 1 or 2 or 3 or 4 or 5 or 6 or 7 or 8 or 9 or 10 or 11 (228159)

13 exp Osteoarthritis/ (59124)

14 Intervertebral Disc Degeneration/ (3918)

15 (degenerat* adj3 (joint* or spin* or hip* or knee* or back or low* back or chang*)).mp. (25233)

16 (osteo-arthriti* adj3 (joint* or spin* or hip* or knee* or back or low* back)).mp. (106)

17 13 or 14 or 15 or 16 (84504)

18 (low* and (backpain or backache)).mp. [mp=title, abstract, original title, name of substance word, subject heading word, keyword heading word, protocol supplementary concept word, rare disease supplementary concept word, unique identifier, synonyms] (1247)

19 (low* back and (pain or ache)).mp. [mp=title, abstract, original title, name of substance word, subject heading word, keyword heading word, protocol supplementary concept word, rare disease supplementary concept word, unique identifier, synonyms] (30025)

20 back pain/ or low back pain/ (36556)

21 ((lumbar or spin* or knee* or hip*) adj3 (pain* or ache*)).mp. (21324)

22 lumbago.mp. (1255)

23 Pain/ and Hip Joint/ (1067)

24 Knee Joint/ and Pain/ (2130)

25 18 or 19 or 20 or 21 or 22 or 23 or 24 (63883)

***************************

Database: Embase <1974 to 2018 January 08>

Search Strategy:

--------------------------------------------------------------------------------

1 health behavior/ (56520)

2 ((health* or wellness or lifestyle or life style) adj3 (behavio?r or coach*)).mp. (72910)

3 directive counseling/ or motivational interviewing/ (3928)

4 (motivation* adj3 (counsel* or interview*)).mp. (6085)

5 (behavio?r adj3 change*).mp. (54187)

6 Motivation/ (89590)

7 ((self or intrinsic* or internal*) adj3 motivat*).mp. (5277)

8 coach* psycholog*.mp. (12)

9 ((trans-theoretic* or transtheoretic*) and model*).mp. (1747)

10 self care/ or self help/ (57900)

11 (self manag* or selfmanag*).mp. (21485)

12 1 or 2 or 3 or 4 or 5 or 6 or 7 or 8 or 9 or 10 or 11 (267814)

13 Osteoarthritis.mp. (117956)

14 intervertebral disk degeneration/ or intervertebral disk disease/ (10431)

15 (degenerat* adj3 (joint* or spin* or hip* or knee* or back or low* back or chang*)).mp. (37331)

16 (osteo-arthriti* adj3 (joint* or spin* or hip* or knee* or back or low* back)).mp. (155)

17 13 or 14 or 15 or 16 (157443)

18 low back pain/ (48511)

19 (low* back and (pain or ache)).mp. (56931)

20 (low* and (backpain or backache)).mp. (13633)

21 ((lumbar or spin* or knee* or hip*) adj3 (pain* or ache*)).mp. (43466)

22 lumbago.mp. (1680)

23 Pain/ and Hip Joint/ (2968)

24 Knee Joint/ and Pain/ (4906)

25 18 or 19 or 20 or 21 or 22 or 23 or 24 (107336)

26 17 or 25 (245644)

27 12 and 26 (2778)

***************************

Database: PsycINFO <1967 to January Week 1 2018>

Search Strategy:

--------------------------------------------------------------------------------

1 health behavior/ or health promotion/ or lifestyle changes/ (44455)

2 health behavior/ or patient compliance/ (24523)

3 ((health or wellness or lifestyle or life) and coach*).mp. [mp=title, abstract, heading word, table of contents, key concepts, original title, tests & measures] (3545)

4 motivational interviewing/ or behavior change/ or readiness to change/ or "stages of change"/ or transtheoretical model/ (14492)

5 behavior change/ or behavior modification/ or change strategies/ or lifestyle changes/ or motivational interviewing/ or readiness to change/ or "stages of change"/ or transtheoretical model/ (25546)

6 ((self or intrinsic* or internal*) adj3 motivat*).mp. (14857)

7 coach* psycholog*.mp. (589)

8 ((trans-theoretic* or transtheoretic*) and model*).mp. [mp=title, abstract, heading word, table of contents, key concepts, original title, tests & measures] (1901)

9 Self care.mp. (10686)

10 (self manag* or selfmanag*).mp. [mp=title, abstract, heading word, table of contents, key concepts, original title, tests & measures] (10404)

11 1 or 2 or 3 or 4 or 5 or 6 or 7 or 8 or 9 or 10 (102878)

12 Osteoarthritis.mp. (1700)

13 Intervertebral Disc Degeneration.mp. (10)

14 (degenerat* adj3 (joint* or spin* or hip* or knee* or back or low* back or chang*)).mp. (1097)

15 (osteoarthriti* and (joint* or spin* or hip* or knee* or back or low* back)).mp. [mp=title, abstract, heading word, table of contents, key concepts, original title, tests & measures] (1035)

16 (degenerat* and hip).mp. [mp=title, abstract, heading word, table of contents, key concepts, original title, tests & measures] (33)

17 12 or 13 or 14 (2772)

18 low back pain.mp. (3194)

19 (low* back and (pain or ache)).mp. [mp=title, abstract, heading word, table of contents, key concepts, original title, tests & measures] (3708)

20 (low* and (backpain or backache)).mp. [mp=title, abstract, heading word, table of contents, key concepts, original title, tests & measures] (46)

21 ((lumbar or spin* or knee* or hip*) and (pain* or ache*)).mp. [mp=title, abstract, heading word, table of contents, key concepts, original title, tests & measures] (10739)

22 lumbago.mp. (29)

23 (Hip joint and pain).mp. [mp=title, abstract, heading word, table of contents, key concepts, original title, tests & measures] (34)

24 (Knee joint and pain).mp. [mp=title, abstract, heading word, table of contents, key concepts, original title, tests & measures] (138)

25 18 or 19 or 20 or 21 or 22 or 23 or 24 (13636)

26 17 or 25 (15696)

27 11 and 26 (364)

***************************

Database: CINAHL

Search Strategy:

--------------------------------------------------------------------------------

S1 (MH "Health Behavior+") (58,521)

S2 (MH "Behavioral Changes") OR (MH "Transtheoretical Stages of Change Model") OR (MH "Health Promoting Behavior (Iowa NOC)") (7,413)

S3 ""health coaching or wellness coaching"" (328)

S4 "health or wellness or lifestyle or life coach" (0)

S5 "lifestyle coach" (4)

S6 (MH "Motivational Interviewing") (1,679)

S7 "motivational interviewing" (2,358)

S8 "self motivation" (128)

S9 "self care" (29,859)

S10 "self management" (7,009)

S11 S1 OR S2 OR S3 OR S4 OR S5 OR S6 OR S7 OR S8 OR S9 OR S10 (94,237)

S12 (MH "Osteoarthritis+") OR (MH "Osteoarthritis, Spine+") OR (MH "Osteoarthritis, Knee") OR (MH "Osteoarthritis, Hip") (12,912)

S13 "osteoarthritis" (15,658)

S14 "Intervertebral Disc Degeneration" (170)

S15 "degenerative joint disease" (265)

S16 "knee joint degeneration" (11)

S17 "knee or hip or back or spine or joint degeneration" (0)

S18 "hip joint degeneration" (2)

S19 "spine degeneration" (22)

S20 "joint degeneration" (180)

S21 "back degeneration" (0)

S22 S12 OR S13 OR S14 OR S15 OR S16 OR S17 OR S18 OR S19 OR S20 OR S21 (16,234)

S23 (MH "Low Back Pain") (11,567)

S24 (MH "Back Pain+") (18,147)

S25 "knee joint pain" (37)

S26 "hip joint pain" (15)

S27 S23 OR S24 OR S25 OR S26 (18,199)

S28 S22 OR S27 (33,951)

S29 S11 AND S28 (1,215)

***************************************

Database: EBM Reviews - Cochrane Central Register of Controlled Trials <December 2017>

Search Strategy:

--------------------------------------------------------------------------------

1 health behavior/ or patient compliance/ (11855)

2 ((health or wellness or lifestyle or life) and coach*).mp. [mp=title, original title, abstract, mesh headings, heading words, keyword] (1203)

3 directive counseling/ or motivational interviewing/ (844)

4 motivation* counsel*.mp. (82)

5 Behavio?r change.mp. (3102)

6 ((self or intrinsic or internal) and motivat*).mp. [mp=title, original title, abstract, mesh headings, heading words, keyword] (3916)

7 ((trans-theoretic* or transtheoretic*) and model*).mp. [mp=title, original title, abstract, mesh headings, heading words, keyword] (304)

8 self care/ (3498)

9 (self manag* or selfmanag*).mp. [mp=title, original title, abstract, mesh headings, heading words, keyword] (3787)

10 1 or 2 or 3 or 4 or 5 or 6 or 7 or 8 or 9 (23577)

11 osteoarthritis/ or osteoarthritis, hip/ or osteoarthritis, knee/ or osteoarthritis, spine/ (4055)

12 Intervertebral Disc Degeneration/ (141)

13 (degenerat* and (joint* or spin* or hip* or knee* or back or low* back or chang*)).mp. [mp=title, original title, abstract, mesh headings, heading words, keyword] (3238)

14 11 or 12 or 13 (7122)

15 Low Back Pain/ (2201)

16 (low* and (backpain or backache)).mp. [mp=title, original title, abstract, mesh headings, heading words, keyword] (1040)

17 (low* back and (pain or ache)).mp. [mp=title, original title, abstract, mesh headings, heading words, keyword] (6119)

18 lumbago.mp. (154)

19 (Hip joint and pain).mp. [mp=title, original title, abstract, mesh headings, heading words, keyword] (324)

20 (Knee joint and pain).mp. [mp=title, original title, abstract, mesh headings, heading words, keyword] (1590)

21 15 or 16 or 17 or 18 or 19 or 20 (8829)

22 14 or 21 (14786)

23 10 and 22 (466)

***************************

Database: Web of Science

Search strategy:

1 TOPIC: ((health OR wellness OR lifestyle OR behaviour) AND coach) ([5,758](http://apps.webofknowledge.com/summary.do?product=WOS&doc=1&qid=42&SID=F4QlKAvqWeXd4OIJvQk&search_mode=GeneralSearch&update_back2search_link_param=yes))

2 TOPIC: (Motivation* interview OR Motivation* counsel* OR directive counsel*) ([17,847](http://apps.webofknowledge.com/summary.do?product=WOS&doc=1&qid=43&SID=F4QlKAvqWeXd4OIJvQk&search_mode=GeneralSearch&update_back2search_link_param=yes))

3 TOPIC: (behavi?r change OR transtheoretical model) ([324,747](http://apps.webofknowledge.com/summary.do?product=WOS&doc=1&qid=44&SID=F4QlKAvqWeXd4OIJvQk&search_mode=GeneralSearch&update_back2search_link_param=yes))

4 TOPIC: (self motivat* OR intrinsic* OR internal* motivat*) ([371,187](http://apps.webofknowledge.com/summary.do?product=WOS&doc=1&qid=45&SID=F4QlKAvqWeXd4OIJvQk&search_mode=GeneralSearch&update_back2search_link_param=yes))

5 TOPIC: (self motivat* OR intrinsic* motivat* OR internal* motivat*) ([64,217](http://apps.webofknowledge.com/summary.do?product=WOS&doc=1&qid=46&SID=F4QlKAvqWeXd4OIJvQk&search_mode=GeneralSearch&update_back2search_link_param=yes))

6 #5 OR #4 OR #3 OR #2 OR #1 ([703,333](http://apps.webofknowledge.com/summary.do?product=WOS&doc=1&qid=47&SID=F4QlKAvqWeXd4OIJvQk&search_mode=CombineSearches&update_back2search_link_param=yes))

7 TOPIC: (osteoarthritis) ([71,378](http://apps.webofknowledge.com/summary.do?product=WOS&doc=1&qid=49&SID=F4QlKAvqWeXd4OIJvQk&search_mode=GeneralSearch&update_back2search_link_param=yes))

8 TOPIC: (lumbar osteoarthritis OR knee osteoarthritis OR hip osteoarthritis) ([42,172](http://apps.webofknowledge.com/summary.do?product=WOS&doc=1&qid=50&SID=F4QlKAvqWeXd4OIJvQk&search_mode=GeneralSearch&update_back2search_link_param=yes))

9 TOPIC: (intervertebral disc degeneration OR intervertebral disc disease) ([6,413](http://apps.webofknowledge.com/summary.do?product=WOS&doc=1&qid=51&SID=F4QlKAvqWeXd4OIJvQk&search_mode=GeneralSearch&update_back2search_link_param=yes))

10 TOPIC: (lumba* pain OR low back pain OR back pain) ([72,009](http://apps.webofknowledge.com/summary.do?product=WOS&doc=1&qid=53&SID=F4QlKAvqWeXd4OIJvQk&search_mode=GeneralSearch&update_back2search_link_param=yes))

11 TOPIC: ((lumba* OR low back OR back) ache) ([422](http://apps.webofknowledge.com/summary.do?product=WOS&doc=1&qid=54&SID=F4QlKAvqWeXd4OIJvQk&search_mode=GeneralSearch&update_back2search_link_param=yes))

12 TOPIC: (hip joint pain OR knee joint pain) ([14,022](http://apps.webofknowledge.com/summary.do?product=WOS&doc=1&qid=55&SID=F4QlKAvqWeXd4OIJvQk&search_mode=GeneralSearch&update_back2search_link_param=yes))

13 #12 OR #11 OR #10 OR #9 OR #8 OR #7 ([150,568](http://apps.webofknowledge.com/summary.do?product=WOS&doc=1&qid=56&SID=F4QlKAvqWeXd4OIJvQk&search_mode=CombineSearches&update_back2search_link_param=yes))

14 #13 AND #6 ([2,166](http://apps.webofknowledge.com/summary.do?product=WOS&doc=1&qid=57&SID=F4QlKAvqWeXd4OIJvQk&search_mode=CombineSearches&update_back2search_link_param=yes))

**APPENDIX 2: Study outcomes relevant to this systematic review**

|  |  |  |  | RESULTS | | | |
| --- | --- | --- | --- | --- | --- | --- | --- |
| Study | Outcome measure | Measurement time points | Participant retention (% of original cohort) | Control group mean (SD) | Intervention group  Mean (SD) | Mean difference (95% confidence interval) | Significance (P value) |
| **Hip/Knee** |  |  |  |  |  |  |  |
| Bennell et al 2017 [9] | Pain NRS (0-10)  *WOMAC function subscale 0-68  AQoL (0.0-1.0)  Physical activity (PASE 0 - >400) | Baseline  6 months  12 months  18 months  Baseline  6 months  12 months  18 months  Baseline  6 months  12 months  18 months  Baseline  6 months  12 months  18 months | 85  81  76  85  81  76  85  81  76  85  81  76 | 5.8 (1.5)  3.8 (2.3)  3.7 (2.2) 4.1 (2.8)  30.3 (10.1)  18.2 (11.7) 17.4 (11.9) 16.4 (11.7)  0.7 (0.1)  0.8 (0.1)  0.8 (0.1)  0.8 (0.2)  151 (78) 158 (63) 166 (77) 162 (70) | 5.6 (1.4)  3.1 (2.2)  3.2 (2.4) 3.6 (2.4  27.3 (11.1) 14.7 (10.6) 13.3 (10.5) 12.2 (10.5**)**  0.7 (0.1)  0.8 (0.1)  0.8 (0.2)  0.8 (0.1)  171 (85) 189 (85) 172 (80) 180 (94) | -0.20 [-0.64, 0.24]  -0.7 (-1.42 – 0.02)  -0.5 (-1.27 – 0.27)  -0.05 (-1.41 – 0.41)  -3 (-6.21- 0.21)  -3.5 (-7.18 – 0.18)  -4.1 (-7.88 - -0.32)  -4.20 (-8.06 - -0.34)  0.0 (-0.03 – 0.03)  0.0 (-0.03 – 0.03)  0.0 (-0.05 – 0.05)  0.0 (-0.06 – 0.06)  20.00 (-4.67, 44.67)  31.00 (6.44, 55.56)  6.00 (-20.39, 32.39)  18.00 (-10.60, 46.60) | 0.37  0.06  0.21  0.28  0.07  0.06  0.04  0.04  1.00  1.00  1.00  1.00  0.11  0.01  0.66  0.22 |
| Brosseau  et al 2012 Part 1 [10] | *Compliance (no. attended walkingsession/no. prescribed) | 0-3 months  3-6 months  6-9 months  9-12 months | 82  75  67  61 | W=0.770(0.299) C =0.652(0.403)  W=0.617(0.410) C =0.535(0.459)  W=0.471(0.418) C =0.528(0.463)  W=0.446(0.441) C =0.490(0.462) | 0.802(0.290)  0.636(0.390)  0.534(0.425)  0.445(0.433) | W/I 0.03 (-0.06,0.13)  C/I 0.15 (0.03, 0.27)  W/I 0.02(-0.11, 0.15)  C/I 0.10 (-0.04, 0.24)  W/I 0.06 (-0.07,0.20)  C/I 0.01 (-0.14, 0.15)  W/I-0.00(-0.14,0.14)  C/I -0.04(-0.19, 0.10) | 0.51  0.01  0.77  0.16  0.37  0.94  0.99  0.55 |
| Brosseau  et al 2012 Part 2 [11] | WOMAC pain subscale (0-20)  WOMAC function subscale (0-68)  SF-36 (SPC)  SF-36 (SMC)  7-day Physical Activity Recall  LTA Walking  Only (mins/day)  LTA Walking  +Other  (mins/day)  ODA Walking  Only  (mins/day)  ODA Walking  +Other  (mins/day) | Baseline  12 months  18 months  Baseline  12 months  18 months  Baseline  12 months  18 months  Baseline  12 months  18 months  Baseline  12 months  18 months  Baseline  12 months  18 months  Baseline  12 months  18 months  Baseline  12 months  18 months | 61  55  61  55  61  55  61  55  61  55  61  55  61  55  61  55 | W=31.15(14.29) C=30.30 (16.47) W=24.65(15.78)  C =25.00(19.44)  W=23.60(15.09)  C=23.50(17.78)  W=28.16(15.41) C =26.89(16.34) W=24.48(13.79) C =25.06(13.53) W=18.20(14.63) C =19.40(17.08)  W=40.516(8.598)  C =41.996(9.100)  W =42.508(9.229)  C =43.464(9.409)  W =42.820(9.240)  C = 45.149(8.930)  W=52.914(10.845)  C =53.556(8.995)  W = 53.819(9.852)  C = 55.162(8.540)  W = 54.476(7.329)  C = 53.101(9.914)  W = 12.09(13.09)  C = 12.45(14.96)  W = 12.22(7.86)  C = 12.68(11.20)  W = 12.20(9.90)  C = 16.88(17.50)  W = 16.18 (21.49)  C = 19.74 (22.87)  W = 15.34 (10.23)  C = 16.01 (14.14)  W = 16.46 (13.17)  C = 24.18 (25.59)  W = 20.75 (25.69)  C = 18.17 (25.98)  W = 17.10 (21.03)  C = 11.05 (8.39)  W = 22.33 (26.10)  C = 12.04 (5.64)  W = 31.31 (36.00)  C = 26.85 (30.88)  W = 22.63 (20.97)  C = 27.99 (37.28)  W = 23.34 (22.40)  C = 26.13 (15.64) | 26.81(14.92)  25.32(15.98)  26.16(17.97)  27.65(18.22)  25.27(15.70)  24.15(17.24)  43.645(8.656)  42.192(10.066)  40.909(11.038)  53.812(8.639)  54.476(7.329)  53.922(9.023)  13.92(16.50)  13.89(12.40)  16.40(18.72)  16.43(18.86)  19.77(15.85)  22.15(21.21)  11.92(12.42)  33.00(70.75)  33.07(39.04)  19.77(25.61)  41.48(61.94)  27.97(33.15) | W/I -4.34 (-9.06, 0.38)  C/I-3.49 (-8.65, 1.67)   | W/I 0.67 (-6.08, 7.42) | | --- |   C/I 0.32 (-7.35, 7.99)  W/I 2.56(-4.50, 9.62)  C/I 2.66(-5.35,10.67)  W/I -0.51(-7.88,6.86)  C/I 0.76 (-6.93, 8.45)  W/I 0.79 (-5.65, 7.23)  C/I 0.21 (-6.31, 6.73)  W/I 5.95 (-0.85, 12.75)  C/I 4.75 (-2.94, 12.44)  W/I 3.13 (0.31, 5.94)  C/I 1.65 (-1.27, 4.57)  W/I -0.32 (-4.38, 3.74)  C/I -1.27 (-5.44, 2.89)  W/I -1.91 (-6.22, 2.40)  C/I -4.24 (-8.67, 0.19)  W/I 0.90 (-2.28, 4.07)  C/I 0.26 (-2.65, 3.16)  W/I 0.66 (-2.99, 4.30)  C/I -0.69 (-4.10, 2.72)  W/I -0.55 (-4.04, 2.93)  C/I 0.82 (-3.41, 5.06)  W/I 1.83 (-3.81, 7.47)  C/I -1.04 (-7.10, 5.02)  W/I 1.67 (-2.98, 6.32)  C/I 1.21 (-4.14, 6.56)  W/I 4.20 (-2.89, 11.29)  C/I -0.48 (-9.29, 8.33)  W/I 0.25 (-6.69, 7.19)  C/I -3.31 (-10.71, 4.09)  W/I 4.43 (-1.46, 10.32)  C/I 3.76 (-2.88, 10.40)  W/I 5.69 (-2.41, 13.79)  C/I -2.03 (-13.24, 9.18)  W/I -8.83 (-18.50,0.84)  C/I -6.25 (-16.12, 3.62)  W/I 15.90 (-28.11,59.91)  C/I 21.95 (-20.11,64.01)  W/I 10.74 (-13.18, 34.66)  C/I 21.03 (0.21, 41.85)  W/I -11.54 (-23.23, 0.15)  C/I -7.08 (-18.04, 3.88)  W/I 18.85 (-7.21, 44.91)  C/I13.49 (-14.65, 41.63)  W/I 4.63 (-10.94, 20.20)  C/I 1.84 (-13.17, 16.85) | 0.07  0.19  0.85  0.94  0.48  0.52  0.89  0.85  0.81  0.95  0.09  0.23  0.03  0.27  0.88  0.55  0.39  0.07  0.59  0.86  0.73  0.69  0.76  0.70  0.52  0.74  0.47  0.66  0.23  0.92  0.94  0.39  0.14  0.27  0.16  0.72  0.10  0.25  0.54  0.29  0.38  0.05  0.05  0.22  0.15  0.33  0.55  0.83 |
| Gilbert et al [26] | WOMAC pain subscale (0-20)  *WOMAC function subscale (0-68)  SF-36 (PCS)  SF-36 (MCS)  Average daily activity (minutes)  Average daily activity moderate-vigorous (minutes) | Baseline  3 months  6 months  12 months  24 months  Baseline  3 months  6 months  12 months  24 months  Baseline  3 months  6 months  12 months  24 months  Baseline  3 months  6 months  12 months  24 months  Baseline  3 months  6 months  12 months  24 months  Baseline  3 months  6 months  12 months  24 months | 80  79  67  48  80  79  67  8  80  79  67  48  80  79  67  48  80  79  67  48  80  79  67  48 | 5.49 (3.42)  6.14 (2.17)  5.49 (2.28)  5.6 (2.56)  4.71 (2.94)  17.42(11.40)  17.80 (6.0)  16.69 (6.59)  16.60 (7.45)  15.33 (8.63)  44.51(8.13)  44.67 (5.85)  44.81 (5.53)  44.32 (6.16)  44.66 (7.32)  54.40(6.84)  54.59 (7.01)  54.05 (7.57)  54.68 (5.53)  52.84 (8.76)  470.50(99.24)  489.94 (66.35)  472.27 (73.34)  474.1 (83.83)  484.14 (89.08)  17.34(21.28)  16.18 (8.52)  15.95 (11.15)  17.74 (10.33)  15.88 (9.76) | 5.88 (3.64)  5.17 (2.42)  5.31 (2.6)  4.76 (2.57)  3.96 (2.21)  18.04(12.41)  16.51 (7.47)  15.13 (7.76)  13.41 (8.06)  12.53 (7.04)  44.96(8.19)  46.03 (5.29)  45.04 (5.50)  46.03 (5.03)  45.44 (5.97)  53.83 (7.94)  53.59 (6.74)  54.32 (6.71)  54.06 (6.23)  54.18 (6.32)  507.30(105.3)  495.31 (75.27)  524.17(153.47)  484.91 (71.43)  472.06 (85.21)  22.73(18.51)  15.63 (8.43)  18.51 (14.40)  15.03 (11.51)  11.4 (14.12) | 0.39 (-0.72, 1.50)  -0.97 (-1.77, -0.17)  -0.18 (-1.05, 0.69)  -0.90 (-1.89, 0.09)  -0.75 (-1.92, 0.42)  0.62 (-3.14, 4.38)  -1.29 (-3.66, 1.08)  -1.56 (-4.13, 1.01)  -3.19 (-6.18, -0.20)  -2.80 (-6.35, 0.75)  0.45 (-2.12, 3.02)  1.36 (-0.60, 3.32)  0.23 (-1.72, 2.18)  1.71 (-0.44, 3.86)  0.78 (-2.23, 3.79)  -0.57 (-2.91, 1.77)  -0.63 (-3.04, 1.78)  0.27 (-2.25, 2.79)  -0.62 (-3.33, 2.09)  1.34 (-2.09, 4.77)  36.80 (4.56, 69.04)  5.37 (-19.46, 30.20)  51.90 (8.31, 95.49)  10.81 (-19.05, 40.67)  -12.08 (-51.56, 27.40)  5.39 (-0.88, 11.66)  -0.55 (-3.52, 2.42)  2.56 (-2.04, 7.16)  -2.71 (-6.92, 1.50)  -4.47 (-10.04, 1.08) | 0.49  0.02  0.69  0.07  0.21  0.75  0.29  0.23  0.04  0.12  0.73  0.17  0.82  0.12  0.61  0.63  0.61  0.83  0.65  0.44  0.03  0.67  0.02  0.48  0.55  0.09  0.72  0.28  0.21  0.12 |
| Hinman et al 2020 [45] | *Pain (NRS 0 - 10)  WOMAC function subscale (0-68)  Physical activity (PASE 0 - >400)  AQol (0.0 – 1.0) | Baseline  6 months  12 months  Baseline  6 months  12 months  Baseline  6 months  12 months  Baseline  6 months  12 months | 94  90  94  90  94  90  94  90 | 6.0 (1.5)  4.2 (2.2)  4.0 (2.3)  27.8 (12.0)  22.0 (12.5)  20.1 (12.5)  163 (81)  172 (99)  152 (87)  0.7 (0.2)  0.7 (0.2)  0.7 (0.2) | 6.0 (1.5)  3.5 (2.1)  3.9 (2.4)  29.3 (10.1)  18.4 (11.3)  18.1 (11.4)  170 (91)  190 (91)  193 (115)  0.7 (0.2)  0.7 (0.2)  0.7 (0.2) | 0.00 (-0.44, 0.44)  -0.70 (-1.36, -0.04)  -0.10 (-0.83, 0.63)  1.50 (-1.78, 4.78)  -3.60 (-7.24, 0.04)  -2.00 (-5.74, 1.74)  7.00 (-18.54, 32.54)  18.00 (-11.02, 47.02)  41.00 (9.34, 72.66)  0.00 (-0.06, 0.06)  0.00 (-0.06, 0.06)  0.00 (-0.06, 0.06) | 1.00  0.04  0.79  0.37  0.05  0.29  0.59  0.22  0.01  1.00  1.00  1.00 |
| Li et AL 2020 [46] | *Physical activity (MPVA in minutes/day)  Pain (KOOS 0-100)  QoL (KOOS knee-related subscale 0-100) | Baseline  13 weeks  26 weeks  39 weeks  Baseline  13 weeks  26 weeks  39 weeks  Baseline  13 weeks  26 weeks  39 weeks | 94  90  86    94  90  86  94  90  86 | DG 71.3 (99.8)  DG 49.4 (63.6)  DG 74.6 (102.1)  DG 54.8 (66.2)  DG 65.1 (13.7)  DG 65.9 (15.6)  DG 74.8 (15.4)  DG 72.8 (13.2)  DG 47.5 (16.0)  DG 46.9 (13.6)  DG 54.7 (14.7)  DG 54.7 (14.7) | IG 31.0 (37.3)  IG 37.7 (30.5)  IG 37.0 (32.3)  IG 34.0 (25.2)  IG 72.6 (13.5)  IG 73.1 (15.3)  IG 72.5 (18.3)  IG 72.1 (19.8)  IG 44.0 (16.0)  IG 48.7 (17.5)  IG 49.4 (15.7)  IG 49.3 (19.1) | NA  13.1 (1.6 to 24.5)  NA  NA  NA  2.5 (−4.2 to 9.5)  NA  NA  NA  1.4 (−5.0 to 7.9)  NA  NA | NA  0.03  NA  NA  NA  0.49  NA  NA  NA  0.66  NA  NA |
| O’Brien et al 2018 [44] | *Pain (NRS 0 -10)  WOMAC – (total disability)  WOMAC – Function subscale 0-68  SF-12 (PCS)  SF-12 (MCS)  Physical activity (AAS) | Baseline  Week 6  Week 26  Baseline  Week 6  Week 26  Baseline  Week 6  Week 26  Baseline  Week 6  Week 26  Baseline  Week 6  Week 26  Baseline  Week 6  Week 26 | 87  73  87  73  85  71  85  71  85  71  88  74 | 6.8 (2.0)  6.3 (1.9)  5.9 (2.8)  48.6 (16.5)  48.2 (18.3)  46.6 (20.3)  34.5 (12.2)  34.3 (13.7)  32.8 (15.1)  31.6 (9.5)  32.3 (9.7)  33.4 (8.9)  51.1 (12.8)  49.1 (12.7)  47.4 (12.3)  100.5 (235.0)  116.8 (204.0)  185.3 (383.6) | 6.9 (1.8)  6.3 (2.3)  6.6 (2.5)  47.9 (17.4)  47.4 (17.9)  49.9 (17.0)  34.9 (12.6)  34.0 (13.8)  36.5 (13.2)  31.7 (10.3)  31.7 (10.9)  29.4 (9.4)  46.9 (14.7)  48.6 (14.8)  53.4 (12.4)  114.7 (361.0)  235.9 (486.2)  179.7 (324.8) | 0.10 (-0.58, 0.78)   - 1. (-0.7 to 0.8)   -0.6 (-1.4 to 0.2)  -0.70 (-6.79, 5.39)  0.8 (-4.4, 5.9)  -2.9 (-8.5, 2.7)  0.9 (-3.0, 4.8)  -2.5 (-6.8, 1.7)  NA  0.7 (-2.2, 3.6)  3.3 (0.2, 6.5)  NA  -1.7 (-5.7, 2.2)  -5.7 (-9.9, -1.5)  NA  -109.8 (-245.1, 25.5)  5.3 (-142.8, 153.4) | NA  0.85  0.12  NA  NA  NA  NA  NA  NA  NA  NA  NA  NA  NA  ≤0.01  NA  NA  NA |
| **LBP** |  |  |  |  |  |  |  |
| Basler et al 2007 [7] | HFAQ (% of normal function)  *Average duration of physical activity (minutes/day) | Baseline  6 weeks  6 months  Baseline  6 weeks  6 months | 92  89  92  89 | 66.3 (19.2)  70.2 (17.9) 68.9 (19.7)  14.11 (15.5) 24.7 (16.3) 25.3 (19.7) | 67.3 (18.9) 73.7 (16.5) 72.5 (20.3)  15.98 (21.1) 29.24 (14.6) 29.63 (24.2) | 1.00 (-4.73, 6.73)  3.50 (-1.68, 8.68)  3.60 (-2.41, 9.61)  1.87 (-3.69, 7.43)  4.54 (-0.47, 9.55)  4.33 (-2.79, 11.45) | 0.73  0.19  0.24  0.51  0.08  0.24 |
| Becker et al 2008 [8] | Days in pain (in past 6 months)  *HFAQ (% of normal function)  Euro-Qol (VAS 0-100)  Physical activity (MET hours/week) | Baseline  6 months  12 months  Baseline  6 months  12 months  Baseline  6 months  12 months  Baseline  6 months  12 months | 91.5  87.9  91.5  87.9  91.5  87.9  91.5  87.9 | GL = 101 (132.02) C = 112 (130.96)  GL= 63.35 (66.69)  C = 80.78 (74.23)  GL= 58.48 (76.2)  C = 71.32 (74.2)    GL = 67.52 (21.42)  C = 65.81 (21.90)  GL = 72.94 (25.17)  C = 70.29 (13.93)  GL = 72.956 (26.46)  C = 71.559 (25.22)  GL = 57.19 (19.9)  C = 55.51 (18.92)  GL = 66.59 (19.24)  C = 66.85 (18.81)  GL = 8.46 (18.87)  C = 67.652 (18.05)  GL = 33.17 (31.74)  C = 37.16 (34.22)  GL = 36.47 (34.13)  C = 33.512 (32.74)  GL 46.429 (35.91) C2 42.883 (35.35) | 103 (123.91)  62.91 (74.83)  61.57 (74.3)  68.74 20.99)  73.94 (24.34)  74.637 (25.39)  58.21 (18.87)  67.54 (17.0)  70.375 (17.23)  34.86 (32.26)  36.294 (33.26)  45.393 (35.57) | GL/C 0 (-17.41, 17.41)  C/I -9.00 (-25.77, 7.77)  GL/C -16.43 (-26.83, - 6.03)  C/I -17.86 (-28.18, -7.55)  GL/C -12.84 (-23.38, -2.30)  C/I -9.76 (-20.20, -0.69)  GL/C 2.93 [0.11, 5.75]  C/I 1.71 [-1.15, 4.57]  GL/C 2.65 (-0.70, 6.01)  C/I 3.65 (0.32, 6.98)  GL/C 1.40 (-2.22, 5.02)  C/I 3.11 (-0.47, 6.70)  GL/C1.68 [-0.88, 4.24]  C/I 2.70 [0.22, 5.18]  GL/C -0.25 (2.86, 2.36)  C/I 0.69 (-1.92,3.30)  GL/C 0.80 (-1.75 to 3.34)  C/I 2.72 (0.19 to 5.26)  GL/C -1.35[-4.02, 1.32]  C/I -0.03 [-2.65, 2.59]  GL/C 2.959 ( -1.628 ,7.545)  C/I 2.781 (-1.784 ,7.347  GL/C 3.546 (-1.452, 8.543)  C/I 2.516 (-2.476,7.495) | 1.00  0.29  0.002  0.001  0.018  0.067    0.12  0.03  0.12  0.03  0.45  0.09  0.20  0.03  0.85  0.60  0.54  0.04  0.32  0.98  0.20  0.23  0.202  0.396 |
| Friedrich  et al 1998 [24] | *Pain intensity (VAS 0-100)  *Disability - Low back outcome scale questionnaire (0-75)  COMPLIANCE:  1. No. of sessions patient attended 2. Compliance post treatment end 3. Weekly training frequency  4. No. of minutes exercised per day  5. Total training time mins | Baseline  ~ 3.5 weeks  4 months  12 months  Baseline  ~ 3.5 weeks  4 months  12 months  4 months  12 months  4 months  12 months  4 months  12 months  4 months  12 months  4 months  12 months | 94  80  74  94  80  74  80  74  80  74  80  74  80  74  80  74 | 54.53 (21.73)  44.0 (27.2)  39.8 (26.6)  41.9 (29.6)  42.8 (13.87)  48.4 (16.4)  51.0 (15.7)  50.9 (18.7)  8.6 (2.1)  NA  10.3 (2.9)  30.1 (20.5)  2.9 (1.1)  3.1 (2.2)  16.1 (10.2)  16.4 (13.0)  748 (668)  1515 (1397) | 50.2 (22.78)  35.9 (25.1)  32.7 (24.3)  26.4 (22.2)  42.5 (14.61)  52.6 (16.3)  57.2 (15.7)  58.9 (12.6)  9.6 (1.1)  NA  10.6 (2.7)  28.8 (18.5)  3.6 (0.5)  4.0 (1.9)  17.9 (8.7)  15.5 (8.6)  917 (656)  2024 (2026) | -4.33 (-13.40, 4.74)  -8.10 (-19.97, 3.77)  -7.10 (-18.01, 3.81)  -15.50 (-27.82, -3.18)  -0.30 (-6.11, 5.51)  4.20 (-3.25, 11.65)  6.20 (-0.52, 12.92)  8.00 (0.50, 15.50)  1.00 (0.30, 1.70)  NA  0.30 (-0.90, 1.50)  -1.30 (-11.27, 8.67)  0.70 (0.33, 1.07)  0.90 (-0.09, 1.89)  169 (-118.05, 456.05)  -0.90 (-6.23, 4.43)  169 (-114.29, 452.29)  508 (-388.25, 1404.25) | 0.35  0.19  0.21  0.02  0.92  0.28  0.08  0.04  0.008  NA  0.63  0.80  0.0003  0.08  0.39  0.74  0.25  0.28 |
| Gardner et al 2019 [25] | *Pain (NRS)  *Disability (QBPDS)  Quality of Life (SF-36) | Baseline  2 months  4 months  12 months  Baseline  2 months  4 months  12 months  Baseline  2 months  4 months  12 months | 100  95  75  100  95  75  100  95  75 | 6.4 (1.5)  5.5 (1.8)  5.5 (2.1)  5.3 (2.0)  41.6 (15.1)  34.0 (15.8)  34.0 (16.9)  31.9 (17.6)  49.8 (19.0)  49.8 (18.9)  50.8 (17.7)  52.4 (18.2) | 6.5 (1.6)  3.2 (1.8)  3.2 (1.9)  3.1 (1.9)  44.3 (12.4)  22.9 (15.6)  21.5 (14.5)  20.3 (16.1)  53.2 (16.1)  65.6 (17.6)  67.7 (17.6)  71.8 (16.3) | 1. (-0.7 to 0.7)   2.3 (1.4 to 3.1)  2.3(1.4 to 3.2)  2.1 (1.2 to 3.1)  −2.6 (−9.0 to 3.7)  11.1 (3.8 to 18.3)  12.9 (5.6 to 20.1)  11.6 (3.6 to 19.5)  −3.4 (-11.5 to 4.7)  −15.8 (−24.2 to –7.4)  −17.7 (−26.0 to –9.5)  −19.5 (−27.9 to–11.0) | NA  <0.05  <0.05  <0.05  NA  <0.05  <0.05  <0.05  NA  <0.05  <0.05  <0.05 |
| Huppe et al 2019 [32] | *Pain (CPGQ 0-100%)  Disability (CPGQ)  *SF-12 (PCS)  *SF -12 (MCS)  Physical activity (days/week at least 10 minutes) | Baseline  24 months  Baseline  24 months  Baseline  24 months  Baseline  24 months  Baseline  24 months | 80  80  80  80  80 | 44.3 (20.4)  41.5 (17.84)  2.2 (2.2)  2.0 (1.6)  40.5 (11.1)  41.0 (8.9)  44.5 (12.1)  46.7 (9.7)  2.5 (1.8)  2.4 (1.6) | 46.3 (19.1)  38.7 (17.4)  2.6 (2.1)  1.6 (1.4)  37.6 (9.2)  43.3 (9.1)  46.5 (12.3)  46.9 (9.8)  2.2 (1.6)  2.7 (1.4) | NA  −0.16 (− 0.34 to 0.03)  NA  −0.24 (− 0.43 to − 0.05)  NA  0.26 (0.07 to 0.45)  NA  0.02 (−0.21 to 0.17)  NA  0.21 (0.03, 0.40) | NA  0.110  NA  0.025  NA  0.007  NA  0.855  NA  0.03 |
| Iles et al 2011 [33] | Modified Oswestry Disability Index (0-100)  *PSFS (0-10) | Baseline  4 weeks  12 weeks  Baseline  4 weeks  12 weeks | 90  87  90  87 | 41 (13)  30 (9)  30 (26)  3.1 (2.3)  5.1 (1.8) 5.2 (3.4) | 40 (20)  22 (17)  14 (17)  3.7 (2.3)  6.5 (2.7) 8.3 (2.1) | -1.00 (-13.07, 11.07)  -8.00 (-18.37, 2.37)  -16.00 (-32.89, 0.89)  0.60 (-1.05, 2.25)  1.40 (-0.34, 3.14)  3.10 (0.93, 5.27) | 0.87  0.14  0.08  0.48  0.12  0.01 |
| Lonsdale  et al 2017 [37] | Pain** intensity (VAS 0-10)  Disability – RMDQ**  PSFS** (0-10)  EurQoL**  Physical activity** (total METs/week)  *Home-based adherence**    *Clinic-based adherence**    *Self-reported Specific adherence to back exercises at home ** | 4 weeks  12 weeks  24 weeks  4 weeks  12 weeks  24 weeks  4 weeks  12 weeks  24 weeks  4 weeks  12 weeks  24 weeks  1 week  4 weeks  12 weeks  24 weeks  Week 1  4 weeks  12 weeks  24 weeks  Baseline  Week 1  4 weeks  12 weeks  24 weeks  Baseline  Week 1  4 weeks  12 weeks  24 weeks | 66  67  80  66  67  80  66  67  80  66  67  80  66  67  80  76  66  67  80  76  66  67  80  76  66  67  80 | -0.88 (2.26) -1.31 (2.36) -1.18 (3.19)  -2.11(4.90)  -2.82(5.77)  -4.09(5.95)  0.81(2.02)  1.44(2.32)  1.76(2.74)  0.24(0.29)  0.25(0.28)  0.24(0.27)  -554.89(-554.89) 330.87(4634.43)  1156.63(4992.22)  -221.11(3171.80)  5.61(1.44)  5.85(1.27)  5.10(1.79)  4.86(1.92)  NA  4.30(0.68)  4.50(0.61)  4.49(0.60)  NA  NA  80.20(22.15)  78.30(27.46)  71.40(24.10)  71.27(26.32) | -0.78 (2.37) -1.53 (2.71) -1.53 (2.78)  -2.23(5.82)  -3.48(5.72)  -4.87(5.86)  1.25(2.21)  2.00(2.47)  2.39(2.99)  0.18(0.28)  0.21(0.27)  0.21(0.24)  -811.17(5936.86)  -682.03(7251.97)  -871.39(6659.47)  -917.82(7313.01)  6.09(1.02)  6.03(1.13)  5.59(1.29)  4.95(1.98)  NA  4.49(0.58)  4.50(0.70)  4.65(0.67)  NA  NA  84.63(21.44)  82.63(21.08)  78.42(27.10)  70.31(30.03) | -0.38 (-1.16 to 0.40)  -0.10 (-0.71 to 0.51)  0.18 (-0.48 to 0.83)  -0.94 (-2.53 to 0.65)  -0.49 (-1.83 to 0.85)  -0.05 (-1.58 to 1.49)  0.40 (-0.22 to 1.01)  0.45 (-0.14 to 1.04)  0.50 (-0.28 to 1.28)  -0.05 (-0.12 to 0.01)  -0.04 (-0.10 to 0.01)  -0.03 (-0.09 to 0.03)  -735.22 (-2166.30 to 695.85)  -729.57 (-2043.57 to 584.42)  -723.93 (-1989.77 to 541.91)  -718.28 (-2012.61 to 576.05)  0.46 (0.16 to 0.77)  0.43 (0.15 to 0.71)  0.39 (0.04 to 0.74)  0.36 (-0.12 to 0.83)  NA  0.09 (-0.16 to 0.33)  0.08 (-0.14 to 0.30)  0.07 (-0.19 to 0.34)  NA  NA  4.47 (-1.70 to 10.64)  3.90 (-0.95 to 8.76)  3.34 (-2.64 to 9.32)  2.77 (-5.87 to 11.42) | 0.34  0.75  0.6  0.25  0.47  0.95  0.21  0.14  0.21  0.12  0.13  0.32  0.31  0.28  0.26  0.28  0.00  0.00  0.03  0.14  NA  0.48  0.48  0.58  NA  NA  0.16  0.11  0.28  0.53 |
| Schaller et al 2017 [52] | Pain (0 – 6)  *Total Physical activity  (MET-min/week)  Workplace physical activity  (MET-min/week)  Leisure physical activity  (MET-min/week) | Baseline  6 months  12 months  Baseline  6 months  12 months  Baseline  6 months  12 months  Baseline  6 months  12 months | NA  NA  NA  NA  NA  NA  NA  NA | 4.4 (±1.0)  NA  NA  4916 (6479)  1821 (3158)  1412 (1301)  3374 (5838)  1310 (3077)  931 (1256)  863 (1339)  247 (286)  255 (306) | 4.4 (1.2)  NA  NA  8455 (8664)  2550 (4037)  1679 (1941)  6228 (8285)  1851 (3922)  1142 (1576)  1187 (2120)  374 (464)  388 (744) | 0.00 (-0.56, 0.56)  NA  NA  NA  NA  NA  NA  NA  NA  NA  NA  NA | 1.00  NA  NA  0.02  0.23 (0.79)  0.30  0.10  0.16  0.65  0.79  0.54  0.89 |
| Thanawat and Nualnetr 2017 [56] | *Pain VAS (0-100)  *Function – Oswestry Disability Questionnaire | Baseline  2 months  5 months  8 months  Baseline  2 months  5 months  8 months | 100  96.8  96  100  96.8  96 | 41.4 (18.2) 28.0 (11.0)  30.4 (7.1)  28.1 (6.5)  28.7 (11.6) 16.6 (8.7)  16.7 (4.8)  15.4 (3.9) | 37.7 (16.6) 27.0 (13.8)  22.8 (7.6)  20.3 (4.7)  24.4 (8.6) 14.8 (4.8)  13.7 (3.4)  11.0 (2.8) | -3.70 (-9.78, 2.38)  -1.00 (-5.37, 3.37)  -7.60 (-10.21, -4.99)  -7.80 (-9.81, -5.79)  -4.30 (-7.86, -0.74)  -1.80 (-4.24, 0.64)  -3.00 (-4.30, -1.70)  -4.40 (-5.61, -3.19) | 0.23  0.65  <0.00001  <0.00001  0.02  0.15  <0.00001  <0.00001 |
| Vong et al 2011 [59] | Pain intensity (VAS 0-10)      RMDQ      SF-36 (PCS)    Exercise compliance No session/day x no days/week | Baseline  session 5  session 10  1-month FU  Baseline  session 5  session 10  1-month FU  Baseline  (No data available after baseline)  Baseline  session 5  session 10  1-month FU | 82.89  96  93  82.89  96  93  82.89  96  93 | 5.3 (2.0)  4.2 (1.8)  3.6 (2.4)  3.9 (2.5)  10.05 (5.54)  8.4 (5.4)  7.2 (5.6)  7.6 (6.4)  63.29(18.39)  NA  6.8 (3.7)  6.2 (3.6)  5.8 (4.1) | 5.3 (2.2)  4.3 (2.0)  3.3 (2.1)  3.1 (2.1)  10.0 (4.31)  7.9 (4.2)  6.3 (4.8)  5.6 (4.5)  67.37(16.84)  NA  12.8 (8.1)  13.9 (8.1)  12.9 (7.2) | 0.00 (-0.95, 0.95)  0.10 (-0.84, 1.04)  -0.30 (-1.44, 0.84)  -0.80 (-2.02, 0.42)  -0.05 (-2.28, 2.18)  -0.50 (-2.88, 1.88)  -0.90 9-3.54, 1.74)  -2.00 (4.93, 0.93)  4.08 (-3.85, 12.01)  NA  6.00 (2.87, 9.13)  7.70 (4.53, 10.87)  7.10 (4.02, 10.18) | 1.00  0.84  0.61  0.21  0.66  0.69  0.51  0.19  0.32  NA  0.0002  <0.00001  <0.00001 |
| Williams et al 2018 [63] | *Pain NRS (0-10)  RMDQ (0 – 24)  SF 12 (PCS)  SF 12 (MCS)  Physical activity (AAS): moderate-vigorous ex in mins/week | Baseline  6 weeks  26 weeks  Baseline  6 weeks  26 weeks  Baseline  6 weeks  26 weeks  Baseline  6 weeks  26 weeks  Baseline  6 weeks  26 weeks | NA  NA  79  58  NA  79  65  NA  79  65  NA  81  65 | 6.8 (1.6)  6.2 (2.1)  6.3 (2.4)  15.8 (5.1) 15.8 (5.1) 14.7 (5.9)  29.2 (9.6)  30.3 (10.6)  30.5 (10.1)  46.1 (13.8)  45.0 (11.6) 44.3 (13.3)  146.7 (504.0)  130.6 (382.1) 148.6 (400.0) | 6.7 (1.8)  6.2 (2.1)  5.8 (2.7)  14.7 (5.2) 14.2 (5.6) 13.9 (6.5)  31.3 (9.2)  31.8 (9.1)  32.1 (10.9)  46.7(13.9) 46.6 (11.0) 46.5 (13.8)  73.9(219.3) 95.8(208.3) 229.2(755.1) | -0.10 (-0.63, 0.43)  0.00 (-0.65, 0.65)  -0.50 (-1.29, 0.29)  -1.10 (-2.70, 0.50)  -1.60 (-3.49, 0.29)  -0.80 (-3.39, 1.79)  2.10 (-0.83, 5.03)  1.50 (-1.94, 4.94)  1.60 (-2.53, 5.73)  0.60 (-3.72, 4.92)  1.60 (-2.36, 5.56)  2.20 (-3.11, 7.51)  -72.80 (-193.37, 47.770  -34.80 (-138.36, 68.76)  80.60 (-166.41,327.61) | 0.71  1.00  0.22  0.18  0.1  0.54  0.16  0.39  0.45  0.79  0.43  0.42  0.24  0.51  0.48 |

**NRS** - numerical rating scale; **W** – walking group; **C** – control group; **I** – intervention group; **WOMAC** – Western Ontario ad McMaster Universities Osteoarthritis Index, function subscale 0 - 68; **AQoL** – Assessment of quality of life 0.0- 1.0; **MPVA**  - Moderate to vigorous physical activity; **DG** – delayed group; **IG** – Immediate group; **KOOS** – Knee Injury and Osteoarthritis Outcome score; **ASS** – Active Australia Survey; **no.** – number; **SP-12/36** – Short Form 12/36; **SPM** - Standard physical component; **SCM** - Standardized Mental Component; **PASE** - Physical Activity Scale for the Elderly, 0-400; **IPAQ** - International Physical Activity Questionnaire; **NA** – not available; **LTA** – Leisure time activities; **ODA** – other domestic activities; **PCS** – physical component scores; **MCS** – mental component scores; **HFAQ** – Hannover functional ability questionnaire; **GL** – guideline training only; **Euro-QoL** – European quality of life scale; **VAS** – visual analogue scale; **MET** – metabolic equivalent; **QBPDS** – Quebec back pain disability scale; **CPGQ** – Chromic pain grade questionnaire; **PSFS** – patient specific functional scale; **RMDQ** – Roland Morris disability questionnaire; **FU** – follow-up

***Primary outcome**

****mean change score**

**Appendix 3: Sensitivity analysis to address the potential that conversion of a 0-10 scale to 0-100 scale moderates the results of the Meta-Analysis for CLBP short-term pain levels**

Figure 1: Pooled effect of intervention compared to control for short-term pain levels for CLBP on NRS 0 – 100


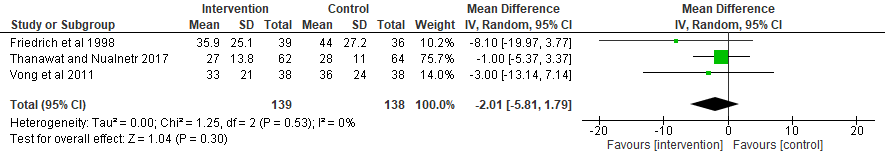


Figure 2: Pooled effect of intervention compared to control for short-term pain levels for CLBP on NRS 0 – 100 with removal of study requiring scale to be converted from 0-10 to 0-100


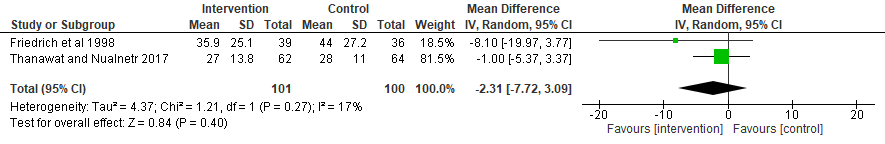

Supplement: pnac099_Supplementary_Data [file pnac099_supplementary_data.zip › pnac099_Supplementary_Data/PRIOR Supplements 1­_2 and 3 CLEAN.docx]
